# Genetic Variation in Base Excision Repair Pathway Genes, Pesticide Exposure, and Prostate Cancer Risk

**DOI:** 10.1289/ehp.1103454

**Published:** 2011-08-02

**Authors:** Kathryn Hughes Barry, Stella Koutros, Sonja I. Berndt, Gabriella Andreotti, Jane A. Hoppin, Dale P. Sandler, Laurie A. Burdette, Meredith Yeager, Laura E. Beane Freeman, Jay H. Lubin, Xiaomei Ma, Tongzhang Zheng, Michael C.R. Alavanja

**Affiliations:** 1 Division of Cancer Epidemiology and Genetics, National Cancer Institute, National Institutes of Health, Department of Health and Human Services, Rockville, Maryland, USA; 2 Yale School of Public Health, New Haven, Connecticut, USA; 3 Epidemiology Branch, National Institute of Environmental Health Sciences, National Institutes of Health, Department of Health and Human Services, Research Triangle Park, North Carolina, USA; 4 Core Genotyping Facility, NCI-Frederick, Frederick, Maryland, USA

**Keywords:** DNA repair, gene–environment interactions, pesticide, polymorphisms, prostate cancer

## Abstract

Background: Previous research indicates increased prostate cancer risk for pesticide applicators and pesticide manufacturing workers. Although underlying mechanisms are unknown, evidence suggests a role of oxidative DNA damage.

Objectives: Because base excision repair (BER) is the predominant pathway involved in repairing oxidative damage, we evaluated interactions between 39 pesticides and 394 tag single-nucleotide polymorphisms (SNPs) for 31 BER genes among 776 prostate cancer cases and 1,444 male controls in a nested case–control study of white Agricultural Health Study (AHS) pesticide applicators.

Methods: We used likelihood ratio tests from logistic regression models to determine *p*-values for interactions between three-level pesticide exposure variables (none/low/high) and SNPs (assuming a dominant model), and the false discovery rate (FDR) multiple comparison adjustment approach.

Results: The interaction between fonofos and rs1983132 in *NEIL3* [nei endonuclease VIII-like 3 (*Escherichia coli*)], which encodes a glycosylase that can initiate BER, was the most significant overall [interaction *p*-value (*p*_interact_) = 9.3 × 10^–6^; FDR-adjusted *p*-value = 0.01]. Fonofos exposure was associated with a monotonic increase in prostate cancer risk among men with CT/TT genotypes for rs1983132 [odds ratios (95% confidence intervals) for low and high use compared with no use were 1.65 (0.91, 3.01) and 3.25 (1.78, 5.92), respectively], whereas fonofos was not associated with prostate cancer risk among men with the CC genotype. Carbofuran and *S*-ethyl dipropylthiocarbamate (EPTC) interacted similarly with rs1983132; however, these interactions did not meet an FDR < 0.2.

Conclusions: Our significant finding regarding fonofos is consistent with previous AHS findings of increased prostate cancer risk with fonofos exposure among those with a family history of prostate cancer. Although requiring replication, our findings suggest a role of BER genetic variation in pesticide-associated prostate cancer risk.

Previous research has demonstrated significantly increased prostate cancer risk for pesticide applicators and pesticide manufacturing workers compared with the general population (Koutros et al. 2010a; Van Maele-Fabry et al. 2006), suggesting a role of pesticides in prostate cancer etiology. Although underlying mechanisms are unknown, a growing body of literature suggests that some pesticides in the organophosphate (OP), organochlorine (OC), carbamate, and pyrethroid insecticide and bipyridyl herbicide classes might induce oxidative stress (Abdollahi et al. 2004; Kisby et al. 2009; Lopez et al. 2007; Mena et al. 2009; Shadnia et al. 2005; Soltaninejad and Abdollahi 2009). Furthermore, several studies (Grover et al. 2003; Kisby et al. 2009; Shadnia et al. 2005; Wong et al. 2008) have observed increased DNA damage with occupational exposure to various groups of pesticides based on the alkaline Comet assay (Singh et al. 1988), which captures some types of damage that can be induced by reactive oxygen species (ROS), such as single-stranded breaks, as well as alkali-labile sites, which can arise during the repair of oxidative DNA base lesions. Studies have also detected increased levels of the 8-hydroxy-2´-deoxyguanosine oxidative DNA lesion in OP-exposed agricultural workers compared with nonagricultural populations (Kisby et al. 2009).

Accumulating DNA damage due to chronic oxidative stress has been proposed as an important mechanism in prostate carcinogenesis (Nelson et al. 2001). Previous research has found increased oxidative DNA lesions in cancerous prostate tissue compared with histologically normal prostate tissue, as well as increasing lesions with increasing age, an important risk factor for prostate cancer (Malins et al. 2001). Some studies have also found altered prostate cancer risk with genetic variation in several genes involved in base excision repair (BER), the predominant pathway involved in repairing oxidative DNA damage (Park et al. 2009). This pathway entails removal of the damaged bases and resulting abasic region, followed by insertion of the correct nucleotides and ligation to seal the gap (Lu et al. 2001). Although genome-wide association studies have not implicated BER gene loci in prostate cancer risk (Eeles et al. 2008; Thomas et al. 2008), these studies have not focused on populations exposed to pesticides or other putative oxidative stress-inducing agents, in which BER genetic variation may be more important.

Given the potential importance of oxidative damage in pesticide-associated prostate cancer risk and the role of the BER pathway in repairing this type of damage, we conducted a nested case–control study of white male pesticide applicators within the Agricultural Health Study (AHS) to evaluate interactions between pesticide exposures and genetic variation in 31 BER genes with respect to prostate cancer. We hypothesized that BER gene variants may modify pesticide-associated prostate cancer risk.

## Materials and Methods

*Study population.* The AHS prostate cancer nested case–control study has been described in detail previously (Koutros et al. 2010b). Briefly, eligible cases were white pesticide applicators who *a*) were diagnosed with prostate cancer between 1993 and 2004 after enrollment in the AHS cohort, *b*) provided a buccal cell sample, and *c*) had no previous history of cancer except nonmelanoma skin cancer. Eligible controls were white male applicators in the cohort who *a*) provided a buccal cell sample, *b*) had no previous history of cancer except nonmelanoma skin cancer, and *c*) were alive at the time of case diagnosis. Previous work in the AHS has demonstrated minimal differences with respect to a variety of characteristics between participants that did and did not provide a buccal cell sample (Engel et al. 2002). Controls were frequency matched 2:1 to cases by date of birth (± 1 year). Based on these inclusion criteria, 841 cases and 1,659 controls were identified. As described previously (Koutros et al. 2010b), exclusions because of insufficient number of available chips (164 controls with the lowest DNA mass), quality control issues [insufficient/poor DNA quality (*n* = 20) or < 90% completion rate for genotyping assays (*n* = 88)], or a genetic background that was inconsistent with European ancestry [< 80% European ancestry using STRUCTURE software, version 2.3.3 (*n* = 3) (Pritchard et al. 2000) or significant deviation from the first two components in principal components analysis (*n* = 5)] resulted in a final sample size of 776 cases and 1,444 controls. All participants provided written informed consent, and the study was approved by the institutional review boards of all participating institutions.

*Exposure assessment.* Information on lifetime use of 50 pesticides was captured in two self-administered questionnaires completed during cohort enrollment (1993–1997). All 2,220 nested case–control study participants completed the first (enrollment) questionnaire, which inquired about ever/never use of the 50 pesticides, as well as duration (years) and frequency (average days per year) of use for a subset of 22 of the pesticides; 1,439 of these men (60.4% of cases and 67.2% of controls) completed the second (take-home) questionnaire, which inquired about use of the remaining 28 pesticides. A previous AHS analysis demonstrated similar characteristics, except for age, between cohort participants who completed the take-home questionnaire and those who did not (Tarone et al. 1997). For each pesticide, we computed total lifetime days of application (number of years × days per year applied) using midpoints of the indicated categories. We also computed an intensity-weighted metric by multiplying the total lifetime days by an intensity score, which was derived from an algorithm based on mixing status, application method, equipment repair, and use of personal protective equipment (Dosemeci et al. 2002) that was recently updated (Coble J, personal communication). For permethrin, we summed exposure variables for crop and animal applications because these were asked about separately. We categorized lifetime days and intensity-weighted lifetime days of application for each pesticide into a three-level, ordinal-valued variable (none/low/high), with low and high categories distinguished by the median among exposed controls. Because of statistical power limitations, we excluded the 10 pesticides with < 10% prevalence among the cases (trichlorfon, ziram, aluminum phosphide, ethylene dibromide, maneb/mancozeb, chlorothalonil, carbon tetrachloride/carbon disulfide, dieldrin, aldicarb, and 2,4,5-trichlorophenoxypropionic acid), leaving 39 available for analysis. All analyses were based on AHS data release version P1REL0712.04 [National Cancer Institute (NCI), Rockville, MD].

*Genotyping and single-nucleotide polymorphism (SNP) selection.* DNA was extracted from buccal cells using the Autopure protocol (Qiagen Inc., Valencia, CA). Genotyping was performed at the NCI Core Genotyping Facility using a custom Infinium® BeadChip assay (iSelect™) from Illumina Inc. (San Diego, CA) as part of an array of 26,512 SNPs in 1,291 candidate genes. Blinded duplicate samples (2%) were included, and SNP concordance ranged from 96% to 100%. Tag SNPs were chosen to cover candidate DNA repair genes for three ancestry populations [Caucasian (CEU), Japanese Tokyo (JPT) + Chinese Beijing (CHB), and Yoruba people of Ibadan, Nigeria (YRI)] in the HapMap Project [data release 20/phase II, National Center for Biotechnology Information (NCBI) Build 36.1 assembly, dbSNPb126 (International HapMap Project 2011)] to allow use of this custom iSelect panel for studies containing different ethnic populations. Tag SNPs were chosen using a modified version of the method described by Carlson et al. (2004) as implemented in the Tagzilla module of the GLU software package, version 1.0b2 (Jacobs 2010). For each candidate gene, SNPs within the region spanning 20 kb 5´ of the start of transcription to 10 kb 3´ of the end of the stop codon were grouped using a binning threshold of *r*^2^ = 0.80, and one tag SNP per bin was selected. Bins were created for each HapMap population, and the optimal tag SNPs were selected such that all three populations were adequately covered at a minimum binning threshold of *r*^2^ = 0.8. Select SNPs previously reported as being potentially functional were also included.

There were 31 BER genes included in the iSelect platform, which were selected based on supplementary information from a review of DNA repair genes (Wood et al. 2005, 2009). Of the 698 tag SNPs selected and genotyped for these genes, 626 remained after quality control exclusions (completion rate < 90% or Hardy-Weinberg equilibrium *p*-value < 1 × 10^–6^). We further restricted SNPs to those with a minor allele frequency (MAF) of ≥ 10% among controls because of limited power for interaction assessments with rarer variants, which resulted in 394 SNPs.

*Statistical analysis.* We used unconditional logistic regression models adjusted for age (< 60, 60–69, ≥ 70 years) and state (Iowa or North Carolina) to estimate main effect odds ratios (ORs) and 95% confidence intervals (CIs) for the 39 pesticides and 394 BER SNPs with prostate cancer risk and to evaluate pesticide × SNP interactions. The addition of family history of prostate cancer and ever/never use of the 5 pesticides most highly correlated with a given pesticide did not alter inference, so these variables were not included in the models.

We examined both intensity-weighted and unweighted pesticide exposure variables, and results were similar; therefore, here we present results only for the intensity-weighted variables. For pesticide main effects analysis and interaction analysis, we used the three-level, ordinal-valued pesticide variables. For the tests of trend with pesticide exposure, we created new variables for each pesticide by assigning participants the value of the median intensity-weighted (or unweighted) lifetime days among controls for their respective exposure category (none/low/high). For SNP main effects analysis, we used variables coded as the number of variant alleles (0, 1, or 2), assuming a log-additive genetic model. To test for interaction, we computed *p*-values from a 1 degree of freedom likelihood ratio test (LRT), using the three-level, ordinal-valued pesticide variables and assuming the dominant genetic model. We used SAS software (version 9.1; SAS Institute Inc., Cary, NC) to estimate ORs for pesticide main effects and stratified effects by genotype, as well as interaction *p*-values (*p*_interact_), and PLINK (Purcell et al. 2007) to estimate ORs for SNP main effects. We evaluated interactions between pesticides and haplotypes for SNPs in linkage disequilibrium (LD) blocks within a gene of interest using generalized linear models, assuming the additive genetic model for haplotypes and treating the most common haplotype as the referent group, using the haplostats package in R (Sinnwell and Schaid 2009). Haplotypes with frequency < 1% were collapsed into a single group. We identified LD blocks using Haploview software (Barrett et al. 2005) based on control data and considering tag SNPs with MAF ≥ 1% among controls. We also used Haploview to compute *r*^2^ values among controls for pairings of SNPs.

We used SAS to calculate false discovery rate (FDR)-adjusted interaction *p*-values with the intensity-weighted pesticide variables (Benjamini and Hochberg 1995). We conducted the FDR analysis by gene (number of comparisons = 39 pesticides × number of tag SNPs for gene) to account for the differing numbers of SNPs by gene. Interactions meeting FDR < 0.2 were considered robust to adjustment for multiple comparisons.

We presented two sets of results for pesticide × SNP interactions. One set encompassed interactions meeting FDR < 0.2. The second set encompassed interactions with a *p*-value < 0.01 for both intensity-weighted and unweighted exposure metrics and a significant increased risk (〈 = 0.05) of prostate cancer following a monotonic pattern with increasing pesticide exposure in one genotype group and no significant association in the other group. We did not focus on interactions involving increased risk with exposure in one genotype group and decreased risk in the other (sometimes referred to as a qualitative interaction) because the biological basis for such a pattern is unclear and a chance effect of the exposure of interest in one of two population subgroups will force this pattern when there is no main effect of the exposure and no confounding (Weiss 2008).

## Results

Nested case–control study participants were representative of prostate cancer cases and cancer-free participants in the cohort with respect to state of residence, applicator type, family history of prostate cancer, and disease characteristics for the cases (Koutros et al. 2010b). Cases were, on average, older at enrollment than men in the cohort as a whole, so their matched controls were older as well. The average age among the nested case–control study participants at the time of enrollment in the cohort was 61 years, compared with 46 years for the cohort. Information on pesticide use in the nested case–control study is available in Supplemental Material, Table 1 (http://dx.doi.org/10.1289/ehp.1103454) .

**Table 1:** Associations between pesticide intensity-weighted lifetime days and prostate cancer.

Pesticide exposure								
None*a*	Low			High		
Pesticide	Ca/Co	Ca/Co	OR (95% CI)*b*	Ca/Co	OR (95% CI)*b*	*p*_trend_*c*
Carbamate insecticides												
Carbaryl		352/633		115/239		0.84 (0.65, 1.09)		102/239		0.63 (0.46, 0.86)		0.01
Carbofuran		433/857		123/224		1.09 (0.85, 1.40)		120/222		1.07 (0.83, 1.38)		0.63
OC insecticides												
Aldrin		481/896		66/157		0.82 (0.59, 1.12)		80/156		0.99 (0.74, 1.34)		0.95
Chlordane		505/888		64/150		0.74 (0.54, 1.01)		65/150		0.74 (0.54, 1.01)		0.04
DDT		373/699		82/222		0.69 (0.52, 0.92)		122/221		1.00 (0.77, 1.30)		0.69
Heptachlor		545/1,003		52/116		0.86 (0.61, 1.22)		47/116		0.78 (0.54, 1.11)		0.15
Lindane		606/1,089		31/87		0.65 (0.43, 1.00)		36/87		0.75 (0.50, 1.12)		0.12
Toxaphene		585/1,084		44/81		1.01 (0.69, 1.48)		35/80		0.75 (0.50, 1.15)		0.19
OP insecticides												
Chlorpyrifos		451/854		166/278		1.14 (0.91, 1.43)		133/277		0.92 (0.72, 1.16)		0.39
Coumaphos		610/1,144		36/66		1.02 (0.67, 1.55)		30/66		0.85 (0.55, 1.33)		0.49
DDVP		603/1,123		44/91		0.90 (0.62, 1.32)		40/91		0.82 (0.56, 1.21)		0.32
Diazinon		513/964		67/123		1.00 (0.73, 1.38)		47/116		0.72 (0.50, 1.03)		0.08
Fonofos		511/992		85/158		1.06 (0.79, 1.42)		92/153		1.19 (0.89, 1.59)		0.25
Malathion		225/399		162/329		0.88 (0.69, 1.13)		152/328		0.80 (0.62, 1.04)		0.13
Parathion		627/1,176		30/43		1.28 (0.79, 2.06)		22/43		0.91 (0.53, 1.54)		0.73
Phorate		462/846		80/175		0.90 (0.66, 1.21)		74/174		0.82 (0.61, 1.11)		0.22
Terbufos		406/803		145/250		1.17 (0.92, 1.50)		131/248		1.07 (0.83, 1.37)		0.74
Pyrethroid insecticide												
Permethrin*d*		576/1,103		78/121		1.24 (0.91, 1.67)		54/121		0.86 (0.61, 1.20)		0.37
Bipyridyl herbicide												
Paraquat		592/1,082		33/86		0.68 (0.45, 1.04)		40/85		0.78 (0.52, 1.17)		0.24
Phosphinic herbicide												
Glyphosate		182/333		276/540		0.93 (0.74, 1.18)		275/533		0.94 (0.74, 1.19)		0.78
Thiocarbamate herbicides												
Butylate		501/903		52/152		0.63 (0.45, 0.88)		72/139		0.94 (0.69, 1.28)		0.72
EPTC		530/1,063		82/120		1.40 (1.03, 1.90)		60/120		1.02 (0.73, 1.42)		0.93
Triazine herbicides												
Atrazine		189/375		274/517		1.07 (0.84, 1.35)		273/516		1.06 (0.84, 1.34)		0.77
Cyanazine		391/698		160/305		0.91 (0.71, 1.16)		129/305		0.73 (0.56, 0.94)		0.02
Metribuzin		433/792		88/188		0.89 (0.67, 1.19)		86/187		0.87 (0.65, 1.15)		0.34
Phenoxy herbicides												
2,4,5-T		500/898		85/153		1.02 (0.77, 1.36)		56/153		0.67 (0.48, 0.93)		0.02
2,4-D		135/218		293/583		0.82 (0.63, 1.06)		295/583		0.82 (0.63, 1.07)		0.50
Benzoic herbicide												
Dicamba		324/573		172/362		0.81 (0.63, 1.04)		176/361		0.82 (0.64, 1.06)		0.29
Chloroacetanilide herbicides												
Alachlor		277/546		200/388		1.02 (0.81, 1.28)		194/387		0.99 (0.79, 1.24)		0.86
Metolachlor		369/712		190/304		1.21 (0.97, 1.52)		119/298		0.77 (0.60, 0.99)		0.02
Dinitroaniline herbicides												
Pendimethalin		474/856		62/170		0.66 (0.48, 0.90)		89/167		0.95 (0.71, 1.25)		0.74
Trifluralin		312/583		177/358		0.93 (0.74, 1.18)		187/356		0.99 (0.78, 1.25)		0.95
Imidazolinone herbicide												
Imazethapyr		411/773		161/263		1.17 (0.91, 1.50)		105/262		0.77 (0.58, 1.01)		0.03
Urea herbicide												
Chlorimuron-ethyl		487/955		78/140		1.11 (0.82, 1.50)		65/139		0.91 (0.66, 1.25)		0.58
Fungicides												
Benomyl		662/1,242		17/35		0.87 (0.48, 1.58)		19/34		0.99 (0.55, 1.76)		0.96
Captan		623/1,144		28/64		0.81 (0.51, 1.29)		33/64		0.94 (0.61, 1.45)		0.79
Metalaxyl		590/1,113		36/76		0.87 (0.57, 1.31)		45/75		1.06 (0.70, 1.61)		0.75
Fumigant												
Methyl bromide		637/1,215		45/101		0.83 (0.56, 1.23)		61/98		1.15 (0.79, 1.68)		0.38
Other												
Petroleum oil/petroleum distillate		488/964		52/103		1.03 (0.72, 1.46)		61/103		1.20 (0.86, 1.68)		0.28
Abbreviations: 2,4,5-T, 2,4,5-trichlorophenoxyacetic acid; 2,4-D, 2,4-dichlorophenoxyacetic acid; Ca, cases; CI, confidence interval; Co, controls; DDT, dichlorodiphenyltrichloroethane; DDVP, dichlorvos; EPTC, *S*-ethyl dipropylthiocarbamate; OC, organochlorine; OP, organophosphate; OR, odds ratio. **a**Referent group for estimated effects of low and high pesticide use. **b**Adjusted for age and state. **c***p*-Value for pesticide trend, adjusted for age and state. **d**Encompasses crop and animal application.												

Similar to observations for the entire AHS cohort (Alavanja et al. 2003), estimated main effects on prostate cancer for the 39 pesticides included in the present study were largely null (Table 1). However, several pesticides exhibited significant inverse trends: carbaryl, chlordane, cyanazine, 2,4,5-trichlorophenoxyacetic acid (2,4,5-T), metolachlor, and imazethapyr (Table 1).

We identified 22 SNPs in 11 genes with *p*_trend_ < 0.05 for main effects on prostate cancer [Table 2; for main effect estimates for all other BER SNPs, see Supplemental Material, Table 2 (http://dx.doi.org/10.1289/ehp.1103454)]. Two SNPs had *p*_trend_ < 0.01: rs3786662 (*p*_trend_ = 0.007), tagged for *PNKP* (polynucleotide kinase 3´-phosphatase), and rs246079 (*p*_trend_ = 0.008), tagged for the uracil-DNA glycosylase gene *UNG*.

**Table 2:** Associations between BER gene SNPs and prostate cancer with *p*_trend_ < 0.05.

SNP	Gene	Function	Location	Variant allele	Chromosome	MAF*a*	OR (95% CI)*b*	*p*_trend_*b*
rs3786662		*PNKP*		Conversion of breaks to ligatable ends		*5120A→T		T		19		0.15		1.25 (1.06, 1.48)		0.007
rs246079		*UNG*		Glycosylase		IVS6-574A→G		G		12		0.41		1.18 (1.04, 1.34)		0.008
rs2184283		*APEX1*		Endonuclease		–17190G→C		C		14		0.33		1.19 (1.04, 1.35)		0.01
rs34260		*UNG*		Glycosylase		*3733G→A		A		12		0.41		1.18 (1.04, 1.33)		0.01
rs246084		*UNG*		Glycosylase		*9235A→G		G		12		0.41		1.16 (1.03, 1.32)		0.02
rs10861152		*TDG*		Glycosylase		IVS2-953G→A		A		12		0.41		0.86 (0.75, 0.97)		0.02
rs322107		*TDG*		Glycosylase		–1484G→A		A		12		0.16		0.81 (0.68, 0.97)		0.02
rs2398668		*NUDT1*		Modulation of nucleotide pools		*7590C→T		T		7		0.36		1.17 (1.03, 1.33)		0.02
rs2270052		*NUDT1*		Modulation of nucleotide pools		*762G→A		A		7		0.35		1.17 (1.02, 1.34)		0.02
rs8113762		*XRCC1*		Ligase-accessory factor		–15466A→G		G		19		0.32		1.16 (1.02, 1.32)		0.03
rs1047490		*TDG*		Glycosylase		–14759A→G		G		12		0.49		1.15 (1.01, 1.30)		0.03
rs17654678		*NTHL1*		Glycosylase		IVS14+216T→G		G		16		0.12		0.80 (0.66, 0.98)		0.03
rs3219476		*MUTYH*		Glycosylase		IVS1-2487C→A		A		1		0.33		1.15 (1.01, 1.31)		0.03
rs174535		*FEN1*		Endonuclease		–11477T→C		C		11		0.36		0.87 (0.76, 0.99)		0.03
rs174532		*FEN1*		Endonuclease		–13959G→A		A		11		0.29		1.15 (1.01, 1.32)		0.03
rs174528		*FEN1*		Endonuclease		–19334T→C		C		11		0.38		0.87 (0.76, 0.99)		0.04
rs427115		*XRCC1*		Ligase-accessory factor		–18586G→A		A		19		0.33		0.87 (0.76, 0.99)		0.04
rs7799006		*NUDT1*		Modulation of nucleotide pools		–4400C→T		T		7		0.35		1.15 (1.01, 1.30)		0.04
rs102275		*FEN1*		Endonuclease		–5030T→C		C		11		0.35		0.87 (0.76, 1.00)		0.04
rs232315		*UNG2*		Glycosylase		–14478C→T		T		5		0.29		1.15 (1.00, 1.31)		0.04
rs4135081		*TDG*		Glycosylase		IVS1-1650A→G		G		12		0.37		1.14 (1.00, 1.29)		0.05
rs7689099		*NEIL3*		Glycosylase		Ex3-64C→G		G		4		0.12		0.82 (0.67, 1.00)		0.05
Abbreviations: CI, confidence interval; MAF, minor allele frequency; OR, odds ratio per allele; SNP, single nucleotide polymorphism. **a**Among controls. **b**Estimated effect of variant allele using an ordinal SNP variable, assuming a log-additive genetic model and adjusting for age and state.																

Fourteen interactions across four genes, *NEIL3* [nei endonuclease VIII-like-3 (*Escherichia coli*)], *DUT* (deoxyuridine triphosphatase), *POLB* [polymerase (DNA directed), beta], and *NTHL1* [nth endonuclease III-like-1 (*E. coli*)], met the FDR < 0.2 criterion (Table 3), including 10 interactions with FDR < 0.01 [interactions between carbaryl and 8 highly correlated SNPs tagging *DUT* (*r*^2^ = 0.61–1.00), one fonofos × *NEIL3* SNP interaction, and one glyphosate × *POLB* SNP interaction]. However, 13 of the 14 combinations were qualitative interactions with a positive association with pesticide exposure among men in one genotype group and an inverse association for men in the other genotype group. The exception was fonofos × *NEIL3* rs1983132. There was a significant monotonic increase in prostate cancer risk with increasing fonofos exposure among men with CT/TT genotypes for rs1983132 [for low compared with no use, OR = 1.65 (95% CI: 0.91, 3.01); for high compared with no use, OR = 3.25 (95% CI: 1.78, 5.92)], but no association among men with the CC genotype [for low compared with no use, OR = 0.93 (95% CI: 0.66, 1.30); for high compared with no use, OR = 0.86 (95% CI: 0.61, 1.21); *p*_interact_ = 9.3 × 10^–6^; FDR-adjusted *p*-value = 0.01] (Table 3). The interaction between fonofos and rs1983132 was the most significant interaction for the *NEIL3* gene and also the most significant of all pesticide × SNP combinations [see Supplemental Material, Table 3 (http://dx.doi.org/10.1289/ehp.1103454) for a summary of all interactions evaluated]. We observed a similar pattern of interaction for fonofos with the moderately correlated *NEIL3* SNP rs17064578 (*r*^2^ = 0.32), although this finding did not meet FDR < 0.2 (Table 4). When we entered both interactions in the model, only the fonofos × rs1983132 interaction remained statistically significant (*p*_interact_ = 8.8 × 10^–4^ and *p*_interact_ = 0.45, respectively). Rs1983132 showed low correlations with other *NEIL3* SNPs (*r*^2^ ≤ 0.15), and analysis of interactions between *NEIL3* haplotypes and fonofos also suggested that rs1983132 might be driving our fonofos × *NEIL3* SNP interaction findings. We observed borderline significant or significant interactions between fonofos and three of four haplotypes that included the variant T allele for rs1983132, including one without the variant C allele for rs17064578, but we did not observe evidence of an interaction with a haplotype that contained the variant allele for rs17064578 and the C allele for rs1983132 (for interaction *p*-values for all *NEIL3* haplotypes, see Supplemental Material, Table 4).

**Table 3:** Pesticide exposure and prostate cancer risk stratified by BER tag SNP genotype for interactions meeting FDR < 0.2.

Pesticide exposure								
None*a*	Low			High		
Pesticide/gene	SNP	Genotype	Ca/Co	Ca/Co	OR (95% CI)*b*	Ca/Co	OR (95% CI)*b*	*p*_interact_*c*	FDR *p*-value*d*
Fonofos																		
*NEIL3*		rs1983132		CC		420/747		62/123		0.93 (0.66, 1.30)		60/128		0.86 (0.61, 1.21)		9.3 × 10^–6^		1.2 × 10^–2^
				CT+TT		91/245		23/35		1.65 (0.91, 3.01)		32/25		3.25 (1.78, 5.92)				
Carbaryl																		
*DUT*		rs11637235		TT		227/358		63/149		0.63 (0.45, 0.89)		45/149		0.35 (0.22, 0.54)		1.3 × 10^–5^		3.1 × 10^–3^
				TC+CC		116/263		51/87		1.32 (0.88, 2.00)		55/85		1.30 (0.81, 2.09)				
*DUT*		rs11631385		AA		264/417		76/173		0.66 (0.48, 0.91)		55/167		0.39 (0.26, 0.58)		2.3 × 10^–5^		3.1 × 10^–3^
				AG+GG		86/214		39/66		1.44 (0.89, 2.31)		47/71		1.65 (0.96, 2.83)				
*DUT*		rs3784619		AA		270/433		81/176		0.71 (0.52, 0.96)		56/173		0.39 (0.26, 0.58)		2.9 × 10^–5^		3.1 × 10^–3^
				AG+GG		82/200		34/63		1.30 (0.79, 2.14)		46/66		1.64 (0.95, 2.82)				
*DUT*		rs13379705		TT		270/436		81/175		0.72 (0.53, 0.98)		56/174		0.40 (0.27, 0.59)		5.3 × 10^–5^		3.9 × 10^–3^
				TC+CC		82/197		34/63		1.28 (0.78, 2.10)		45/65		1.60 (0.92, 2.77)				
*DUT*		rs16960758		TT		271/433		79/177		0.68 (0.50, 0.93)		59/173		0.41 (0.28, 0.60)		9.3 × 10^–5^		5.1 × 10^–3^
				TC+CC		79/195		36/61		1.44 (0.88, 2.36)		43/66		1.63 (0.93, 2.84)				
*DUT*		rs8037626		AA		265/429		79/173		0.71 (0.52, 0.97)		58/170		0.42 (0.28, 0.62)		1.0 × 10^–4^		5.1 × 10^–3^
				AG+GG		79/191		33/60		1.30 (0.78, 2.15)		44/63		1.65 (0.94, 2.91)				
*DUT*		rs12441867		CC		266/428		80/173		0.71 (0.52, 0.97)		56/170		0.41 (0.27, 0.60)		1.2 × 10^–4^		5.1 × 10^–3^
				CT+TT		86/204		35/65		1.26 (0.77, 2.06)		46/69		1.52 (0.88, 2.62)				
*DUT*		rs3784621		TT		253/407		77/169		0.70 (0.51, 0.96)		55/164		0.41 (0.27, 0.61)		1.3 × 10^–4^		5.1 × 10^–3^
				TC+CC		87/210		37/68		1.28 (0.79, 2.06)		45/69		1.51 (0.88, 2.59)				
Glyphosate																		
*POLB*		rs10958713		CC		69/164		110/223		1.17 (0.81, 1.69)		125/189		1.54 (1.06, 2.23)		2.2 × 10^–4^		8.2 × 10^–3^
				CT+TT		113/169		167/316		0.79 (0.58, 1.07)		149/342		0.65 (0.47, 0.89)				
DDVP																		
*NTHL1*		rs8063461		GG		229/405		8/33		0.45 (0.20, 0.99)		9/42		0.40 (0.19, 0.85)		7.0 × 10^–4^		1.6 × 10^–1^
				GA+AA		369/712		36/56		1.21 (0.78, 1.89)		30/48		1.18 (0.74, 1.91)				
Terbufos																		
*NTHL1*		rs17654678		TT		312/627		118/185		1.35 (1.03, 1.78)		114/178		1.35 (1.02, 1.78)		7.4 × 10^–4^		1.6 × 10^–1^
				TG+GG		88/157		26/62		0.67 (0.39, 1.17)		15/61		0.39 (0.21, 0.74)				
Malathion																		
*DUT*		rs11637235		TT		141/226		99/189		0.84 (0.60, 1.16)		77/203		0.60 (0.42, 0.84)		3.8 × 10^–3^		1.2 × 10^–1^
				TC+CC		78/166		60/136		0.95 (0.63, 1.44)		73/117		1.29 (0.86, 1.93)				
Diazinon																		
*DUT*		rs11637235		TT		316/559		30/82		0.63 (0.40, 0.98)		22/67		0.53 (0.31, 0.88)		3.9 × 10^–3^		1.2 × 10^–1^
				TC+CC		185/386		36/41		1.81 (1.11, 2.93)		25/47		1.06 (0.63, 1.80)				
Abbreviations: Ca, cases; CI, confidence interval; Co, controls; DDVP, dichlorvos; FDR, false discovery rate; OR, odds ratio; SNP, single nucleotide polymorphism. **a**Referent group for estimated effects of low and high pesticide use. **b**Adjusted for age and state. **c***p*-Value for interaction from LRT, treating pesticide exposure variables as ordinal variables, assuming the dominant genetic model, and adjusting for age and state. **d**FDR-adjusted interaction *p*-value.																		

**Table 4:** Pesticide exposure and prostate cancer risk stratified by BER tag SNP genotype for interactions meeting *p*_interact_ and stratified pattern criteria.

Pesticide exposure								
None*a*	Low			High		
Pesticide/gene	SNP	Genotype	Ca/Co	Ca/Co	OR (95% CI)*b*	Ca/Co	OR (95% CI)*b*	*p*_interact_*c*	FDR *p*-value*d*
Fonofos																		
*NEIL3*		rs1983132		CC		420/747		62/123		0.93 (0.66, 1.30)		60/128		0.86 (0.61, 1.21)		9.3 × 10^–6^		0.01
				CT+TT		91/245		23/35		1.65 (0.91, 3.01)		32/25		3.25 (1.78, 5.92)				
*NEIL3*		rs17064578		TT		413/763		63/116		0.99 (0.71, 1.39)		65/131		0.90 (0.65, 1.26)		2.8 × 10^–3^		0.51
				TC+CC		91/213		21/40		1.44 (0.78, 2.66)		24/19		3.52 (1.78, 6.95)				
*XRCC1*		rs939460		GG		325/670		62/103		1.18 (0.83, 1.67)		68/86		1.55 (1.08, 2.21)		6.0 × 10^–4^		0.30
				GA+AA		186/322		23/55		0.83 (0.49, 1.42)		24/67		0.72 (0.43, 1.21)				
*XRCC1*		rs2682587		CC		322/665		62/103		1.16 (0.82, 1.65)		66/87		1.46 (1.02, 2.10)		2.4 × 10^–3^		0.30
				CA+AA		188/327		23/55		0.85 (0.50, 1.45)		26/66		0.81 (0.49, 1.34)				
Terbufos																		
*TDG*		rs812498		TT		283/485		90/160		0.99 (0.73, 1.34)		79/168		0.82 (0.60, 1.12)		1.1 × 10^–3^		0.24
				TC+CC		120/306		53/87		1.56 (1.03, 2.36)		51/71		1.86 (1.22, 2.84)				
*TDG*		rs322107		GG		315/550		100/178		1.00 (0.75, 1.33)		92/189		0.86 (0.64, 1.15)		3.5 × 10^–3^		0.24
				GA+AA		91/253		45/72		1.77 (1.12, 2.79)		37/58		1.82 (1.12, 2.96)				
*LIG1*		rs3786763		GG		327/608		111/196		1.08 (0.82, 1.42)		94/206		0.87 (0.65, 1.15)		8.7 × 10^–4^		0.51
				GA+AA		78/194		34/54		1.51 (0.89, 2.55)		37/40		2.32 (1.37, 3.92)				
*LIG1*		rs10407902		CC		323/590		109/193		1.06 (0.80, 1.39)		92/199		0.86 (0.65, 1.15)		1.7 × 10^–3^		0.51
				CG+GG		76/199		33/56		1.51 (0.89, 2.56)		38/47		2.16 (1.29, 3.61)				
*LIG1*		rs3730872		GG		336/618		116/202		1.08 (0.82, 1.41)		97/206		0.88 (0.67, 1.17)		2.0 × 10^–3^		0.51
				GA+AA		67/176		29/44		1.64 (0.93, 2.90)		32/38		2.20 (1.26, 3.83)				
*LIG1*		rs3730912		GG		327/606		112/195		1.09 (0.82, 1.43)		94/203		0.87 (0.66, 1.16)		3.3 × 10^–3^		0.64
				GT+TT		79/197		33/55		1.45 (0.85, 2.45)		37/45		2.09 (1.25, 3.50)				
*LIG1*		rs274883		AA		293/540		98/175		1.05 (0.78, 1.40)		81/179		0.84 (0.62, 1.15)		5.9 × 10^–3^		0.93
				AG+GG		112/263		47/75		1.46 (0.93, 2.29)		50/69		1.75 (1.13, 2.70)				
Carbofuran																		
*NEIL3*		rs1983132		CC		351/642		98/174		1.05 (0.79, 1.39)		83/177		0.86 (0.64, 1.15)		2.8 × 10^–3^		0.51
				CT+TT		82/215		25/50		1.22 (0.70, 2.10)		37/45		2.28 (1.37, 3.81)				
EPTC																		
*NEIL3*		rs1983132		CC		431/806		63/97		1.28 (0.91, 1.81)		37/96		0.76 (0.51, 1.13)		8.3 × 10^–4^		0.37
				CT+TT		99/257		19/23		1.92 (0.99, 3.72)		23/24		2.33 (1.25, 4.34)				
Atrazine																		
*POLE*		rs5744897		CC		155/282		215/406		0.98 (0.75, 1.28)		203/423		0.89 (0.68, 1.16)		9.6 × 10^–4^		0.40
				CT+TT		32/93		58/111		1.51 (0.89, 2.54)		70/91		2.24 (1.33, 3.77)				
*POLE*		rs4883582		CC		152/272		209/390		0.99 (0.75, 1.29)		201/416		0.89 (0.68, 1.16)		2.2 × 10^–3^		0.43
				CA+AA		37/103		65/127		1.37 (0.84, 2.24)		72/100		1.94 (1.19, 3.18)				
Abbreviations: Ca, cases; CI, confidence interval; Co, controls; EPTC, *S*-ethyl dipropylthiocarbamate; FDR, false discovery rate; OR, odds ratio; SNP, single nucleotide polymorphism. **a**Referent group for estimated effects of low and high pesticide use. **b**Adjusted for age and state. **c***p*-Value for interaction from LRT, treating pesticide exposure variables as ordinal variables, assuming the dominant genetic model, and adjusting for age and state. **d**FDR-adjusted interaction *p*-value.																		

Table 4 presents pesticide associations with prostate cancer stratified by genotype for interactions with a *p*-value < 0.01 for both intensity-weighted and unweighted pesticide exposure metrics and a significant monotonic increase in prostate cancer risk with increasing pesticide exposure in one genotype group and no significant association in the other. The results for fonofos × rs1983132 are repeated in Table 4 because the interaction met the criteria described above, in addition to having an FDR < 0.2; otherwise, FDR values were > 0.2 for interactions in Table 4. In addition to interacting with fonofos, *NEIL3* rs1983132 interacted with carbofuran and *S*-ethyl dipropylthiocarbamate (EPTC) such that each pesticide was associated with prostate cancer among men with CT/TT genotypes for this locus [for high use compared with no use, OR = 2.28 (95% CI: 1.37, 3.81) for carbofuran and OR = 2.33 (95% CI: 1.25, 4.34) for EPTC], whereas neither pesticide was associated with prostate cancer among men with the CC genotype (Table 4). Fonofos, carbofuran, and EPTC exposures were moderately correlated (rho ≤ 0.25 for intensity-weighted lifetime days). When we considered joint effects of fonofos, carbofuran, and EPTC exposure by rs1983132 genotype (data not shown), we estimated an OR of 4.33 (95% CI: 2.36, 7.93) for exposure to two or more of these pesticides (compared with no exposure to any of the three pesticides) among men with CT/TT genotypes, but we did not observe evidence of an association among men with the CC genotype (OR = 0.82; 95% CI: 0.59, 1.14).

Other interactions that met the criteria described above included interactions between fonofos, terbufos, and atrazine and correlated SNPs within *XRCC1* (X-ray repair complementing defective repair in Chinese hamster cells 1; *r*^2^ = 0.98), *TDG* (thymine-DNA glycosylase; *r*^2^ = 0.74), *LIG1* (ligase I, DNA, ATP-dependent; five SNPs with *r*^2^ = 0.50–0.96), and *POLE* [polymerase (DNA directed), epsilon; *r*^2^ = 0.88] (Table 4).When we included the two terbufos × *TDG* SNP interactions in the same model, neither achieved statistical significance. However, analysis of interactions between *TDG* haplotypes and terbufos suggested that the *TDG* findings might be driven by rs322107, which also had a significant estimated main effect (*p*_trend_ = 0.02 from Table 2). We observed a significant interaction between terbufos and the *TDG* haplotype that included variant alleles for both rs812498 and rs322107 (C and A, respectively) but did not estimate a significant interaction with the haplotype that contained the variant allele for rs812498 and the wild-type allele for rs322107 [for interaction *p*-values for all *TDG* haplotypes, see Supplemental Material, Table 5 (http://dx.doi.org/10.1289/ehp.1103454)]. When we included the five terbufos × *LIG1* SNP interactions in a single model, terbufos × *LIG1* rs3786763 remained borderline significant (*p*_interact_ = 0.06). Neither the *XRCC1* SNPs nor the *POLE* SNPs could be modeled together because of their high correlations.

## Discussion

Our study is the first to evaluate interactions between pesticide exposures and genetic variation in BER pathway genes with prostate cancer. We observed 14 interactions that were robust to multiple comparison adjustment (FDR < 0.2; Table 3); however, all but one were the result of a positive association in one genotype group and an inverse association in the other (i.e., qualitative interactions that were likely to have occurred by chance). We also presented a second set of results for interactions with *p* < 0.01 for both intensity-weighted and unweighted pesticide exposure metrics, and a significant monotonic increase in prostate cancer risk with increasing exposure in one genotype group and no significant association in the other (Table 4). The only interaction identified through both approaches was fonofos × *NEIL3* rs1983132, which was also the most significant of all interactions evaluated among the 39 pesticides and 394 SNPs.

Fonofos (an OP insecticide) interacted similarly with two moderately correlated *NEIL3* promoter region SNPs, rs1983132 and rs17064578. *NEIL3* encodes a glycosylase enzyme that can initiate BER by recognizing and cleaving damaged bases and introducing a DNA strand break, and thus plays a critical role in this repair pathway. Based on inclusion of both interactions in the model and analysis of *NEIL3* haplotype interactions with fonofos, the associations appeared to be driven by rs1983132. However, the functional significance of this polymorphism is unknown, and it is possible that another variant in LD with rs1983132 that was not examined could be driving our results. Notably, carbofuran (a carbamate insecticide) and EPTC (a thiocarbamate herbicide) showed similar patterns of interaction with rs1983132, although these interactions were weaker and did not remain significant after adjustment for multiple comparisons. The risk of prostate cancer associated with exposure to fonofos, carbofuran, or EPTC alone among men with CT/TT genotypes for rs1983132 appeared to be increased for those exposed to two or more of these pesticides. However, because of relatively wide and overlapping CIs, it is unclear whether the joint effect of these pesticides was driven by fonofos alone.

Lending plausibility to our fonofos × *NEIL3* rs1983132 interaction finding, *in vitro*, experimental animal, and human biomonitoring studies suggest that some OP insecticides might induce oxidative stress and related DNA damage (Kisby et al. 2009; Shadnia et al. 2005; Soltaninejad and Abdollahi 2009). Studies have implicated a role of oxidative stress in OP-induced acute renal tubular necrosis (Poovala et al. 1999), and it has been proposed that oxidative stress might also contribute to OP effects on chronic health outcomes, such as cancers (Mena et al. 2009). There is limited evidence for fonofos genotoxicity based on standard *in vitro* assays (Garrett et al. 1986; Gentile et al. 1982); however, to our knowledge, fonofos has not been specifically examined in relation to indicators of oxidative stress/damage. Although the registrant for fonofos voluntarily canceled the chemical’s registration in 1998 (U.S. Environmental Protection Agency 1999), fonofos was used by about 25% of the nested case–control study participants and thus may have contributed to prostate cancer risk in our study population. Supporting our *NEIL3* interaction finding, fonofos has previously been associated with prostate cancer in the AHS among participants with a family history of prostate cancer (Alavanja et al. 2003; Mahajan et al. 2006), which suggested a role of genetic susceptibility to carcinogenic effects of this chemical.

There is also some plausibility for our interaction findings between carbofuran and EPTC and *NEIL3* rs1983132. Human biomonitoring studies have suggested increased oxidative stress for workers exposed to carbamate insecticides (Lopez et al. 2007; Prakasam et al. 2001). In addition, some, but not all, *in vitro* and animal studies have found increased genetic damage (e.g., mutations) with exposure to carbofuran or products of its nitrosation (Chauhan et al. 2000; Gentile et al. 1982; Hour et al. 1998; Yoon et al. 2001). EPTC metabolites have also been associated with increased DNA damage *in vitro* (Calderón-Segura et al. 2007).

We did not observe highly significant BER SNP main effects in our study. Only two SNPs had a *p*_trend_ < 0.01. These included rs3786662, located 3´ of the BER gene *PNKP* in *PTOV1* (prostate tumor overexpressed 1), which is not part of the BER pathway, and rs246079, located in an intronic region of *UNG* but also tagged for *ALKBH2* [alkB, alkylation repair homolog 2 (*E. coli*)], which is involved in the direct reversal of DNA damage but not BER.

We did not observe main effects or notable interactions for *XRCC1* R399Q (rs25487), *PARP1* [poly (ADP-ribose) polymerase 1] *V762A* (rs1136410), or *OGG1* (8-oxoguanine DNA glycosylase) S326C (rs1052133), although some previous studies have observed phenotypic changes and altered prostate cancer susceptibility with genetic variation at these loci (Park et al. 2009). However, the functional impact of variation at these loci is not fully understood, and it is possible that these SNPs are not important in pesticide-associated prostate cancer risk.

We also did not observe notable interactions between BER SNPs and pesticides in the bipyridyl herbicide, pyrethroid, or OC insecticide classes, despite evidence that some pesticides in these classes might induce oxidative stress (Abdollahi et al. 2004). Although these may be true negative findings, the relatively low prevalence of these pesticides and the likelihood of lower OC exposures in our study population compared with earlier studies, given removal of OCs from the market beginning in the 1970s, might have contributed to our results.

Although there is plausibility for a role of oxidative stress in pesticide-associated carcinogenesis, alternate explanations for our results warrant consideration. Although the BER pathway is the predominant pathway involved in repairing oxidative DNA lesions (Lu et al. 2001), this pathway is also involved in repairing other types of DNA lesions with minimal helix-distorting effect, as well as single-stranded breaks, which could arise from causes other than ROS-induced damage (Lu et al. 2001; Weinberg 2007). It is also possible that our results might be due to chance; however, we took several steps to help reduce false-positive results in our study. We used the FDR method to adjust interaction *p*-values for multiple comparisons. Additionally, we highlighted interactions with a significant monotonic increase in prostate cancer risk with increasing exposure in one genotype group and no significant association in the other. However, we recognize that by focusing on this subset of interaction findings, we might have missed some true positive results among our remaining findings.

Our study was limited in power, and we may have missed some interactions by excluding SNPs with MAF < 10% because of power concerns. Numbers of participants often became small when stratifying by genotype, particularly for the homozygous variant group. We selected the dominant genetic model to help reduce this problem, although this choice could have resulted in a loss of power if another genetic model was more appropriate. Additionally, there were insufficient case numbers to evaluate interactions by prostate cancer stage or grade. However, to our knowledge, no other study has greater power to evaluate pesticide–gene interactions for individual pesticides with prostate cancer.

Our study also has several strengths. We were able to evaluate individual pesticides from a range of chemical and functional classes, which is preferable over grouped evaluation given previous AHS findings suggesting heterogeneity of effect for pesticides within a chemical class (Weichenthal et al. 2010). Furthermore, self-reported pesticide information in the AHS has been demonstrated to be reliable and consistent with the dates of introduction to the market (Blair et al. 2002; Hoppin et al. 2002). We focused our analyses on the intensity-weighted exposure metric, which incorporates an intensity score that has shown moderate correlation with biomarkers of pesticide exposure in postapplication urine samples (Thomas et al. 2010). Additionally, availability of genotyping data for a large number of tag SNPs across the BER pathway allowed us to comprehensively explore the hypothesis that BER genetic variation might modify pesticide-associated prostate cancer risk.

## Conclusions

In this nested case–control study of white male pesticide applicators within the AHS cohort, we observed notable interactions between several pesticides and BER gene variants with respect to prostate cancer. However, only fonofos × *NEIL3* rs1983132 showed an interaction fitting an expected biological pattern that remained significant after adjustment for multiple comparisons. Although we cannot exclude the role of chance in our findings, our interaction results are consistent with a pesticide mechanism of effect involving oxidative stress. Additional studies among pesticide-exposed populations are needed to replicate our findings and to continue to explore mechanisms underlying pesticide associations with cancer.

## Supplemental Material

(381 KB) PDF
