# Supplementary material for: Genetic Variation in Base Excision Repair Pathway Genes, Pesticide Exposure, and Prostate Cancer Risk
Source: Environ Health Perspect. 2011 Aug 2;119(12):1726–32. doi: 10.1289/ehp.1103454 (PMC3261977; doi:10.1289/ehp.1103454)
Supplement: (381 KB) PDF [file ehp.1103454.s001.pdf]

Supplemental Material

Genetic Variation in Base Excision Repair Pathway Genes, Pesticide Exposure, and Prostate Cancer Risk

Kathryn Hughes Barry,[1,2] Stella Koutros,[1] Sonja I. Berndt,[1] Gabriella Andreotti,[1] Jane A. Hoppin,[3] Dale P. Sandler,[3] Laurie A. Burdette,[4] Meredith Yeager,[1,4] Laura E. Beane Freeman,[1] Jay H. Lubin,[1] Xiaomei Ma,[2] Tongzhang Zheng,[2] Michael C.R. Alavanja[1]

[1]Division of Cancer Epidemiology and Genetics, National Cancer Institute, National Institutes of Health, Rockville, MD.

[2]Yale School of Public Health, New Haven, CT.

[3]Epidemiology Branch, National Institute of Environmental Health Sciences, National Institutes of Health, Research Triangle Park, NC.

[4]Core Genotyping Facility, NCI-Frederick, SAIC-Frederick Inc., Frederick, MD.

TABLE OF CONTENTS

Supplemental Material, Table 1. List of pesticides assessed during the enrollment phase of the Agricultural Health Study, prevalence of use among the prostate cancer nested case-control study participants, and median cutpoints to define low and high exposure for unweighted and intensity-weighted metrics, respectively……………………………………………………………………………………3

Supplemental Material, Table 2. BER gene SNPs that showed no effect on prostate cancer (p_{trend}≥0.05) in the Agricultural Health Study……………………………………………………………………..………6

Supplemental Material, Table 3. BER genes included in the iSelect platform and the number of SNPs evaluated, interactions meeting FDR<0.2, and the most significant interaction by gene in the Agricultural Health Study…………………………………………………………………..…………………….20

Supplemental Material, Table 4. Interaction between *NEIL3* haplotypes and fonofos in the Agricultural Health Study…………………………………………………………………..……………………..23

Supplemental Material, Table 5. Interaction between *TDG* haplotypes and terbufos in the Agricultural Health Study………………………………………………………………………………………………24

References……………………………………………………………………………………………26

Supplemental Material, Table 1. List of pesticides assessed during the enrollment phase of the Agricultural Health Study, prevalence of use among the prostate cancer nested case-control study participants, and median cutpoints to define low and high exposure for unweighted and intensity-weighted metrics, respectively.

Chemical/functional class	Pesticide[a,b]	Prevalence of use (% Ever[c]) Cases n (%)	Control n (%)	Median lifetime days[d]	Median intensity-weighted lifetime days[d]
Carbamate insecticide	Aldicarb[b]	56 (8)	97 (7)	24.5	1,079.2
Carbamate insecticide	Carbaryl[b]	395 (53)	767 (55)	24.5	1,040.6
Carbamate insecticide	Carbofuran[a]	261 (38)	461 (35)	24.5	720
Pyrethroid insecticide	Permethrin (animal or crop application)[a]	139 (20)	260 (20)	24.5	810.9
OC insecticide	Aldrin[b]	252 (34)	465 (34)	24.5	590.6
OC insecticide	Chlordane[b]	230 (31)	486 (35)	8.8	402.1
OC insecticide	Dichlorodiphenyltrichloroethane (DDT)[b]	375 (50)	684 (49)	20	612.5
OC insecticide	Dieldrin[b]	66 (9)	151 (11)	8.8	276.5
OC insecticide	Heptachlor[b]	187 (26)	353 (26)	20	572.0
OC insecticide	Lindane[b]	124 (17)	276 (20)	20	724
OC insecticide	Toxaphene[b]	144 (20)	272 (20)	20	780
OP insecticide	Chlorpyrifos[a]	306 (40)	571 (40)	24.5	992.3
OP insecticide	Coumaphos[a]	68 (10)	138 (11)	14.4	737.5
OP insecticide	Diazinon[b]	220 (30)	409 (30)	24.5	882
OP insecticide	Dichlorvos (DDVP)[a]	87 (13)	190 (14)	38.8	1,169
OP insecticide	Fonofos[a]	183 (26)	332 (25)	24.5	882
OP insecticide	Malathion[b]	526 (70)	999 (71)	20	892.5
OP insecticide	Parathion[b]	102 (14)	181 (13)	22.3	869.8
OP insecticide	Phorate[b]	271 (37)	521 (38)	24.5	661.5
OP insecticide	Terbufos[a]	291 (42)	523 (39)	38.8	1,274
OP insecticide	Trichlorfon[a]	5 (0.7)	7 (0.5)	8.8	196.9
Bipyridyl herbicide	Paraquat[b]	135 (19)	298 (22)	8.8	385
Phosphinic herbicide	Glyphosate[a]	573 (76)	1098 (77)	24.5	1,344

Supplemental Material, Table 1 (cont.)

Chemical/functional class	Pesticide[a,b]	Prevalence of use (% Ever[c]) Cases n (%)	Control n (%)	Median lifetime days[d]	Median intensity-weighted lifetime days[d]
Thiocarbamate herbicide	Butylate[b]	227 (31)	463 (34)	24.5	882
Thiocarbamate herbicide	S-ethyl dipropylthiocarbamate (EPTC)[a]	150 (22)	250 (19)	24.5	782
Triazine herbicide	Atrazine[a]	567 (75)	1056 (74)	63.8	2,520
Triazine herbicide	Cyanazine[a]	304 (44)	633 (48)	24.5	1,128.8
Triazine herbicide	Metribuzin[b]	295 (41)	579 (42)	20	630
Phenoxy herbicide	2,4,5-trichlorophenoxyacetic acid (2,4,5-T)[b]	229 (31)	469 (34)	8.8	598.8
Phenoxy herbicide	2,4,5-trichlorophenoxypropionic acid (2,4,5-TP)[b]	64 (9)	141 (10)	22.3	851
Phenoxy herbicide	2,4-dichlorophenoxyacetic acid (2,4-D)[a]	617 (82)	1208 (85)	87.5	3,658.4
Benzoic herbicide	Dicamba[a]	360 (53)	757 (57)	24.5	1,053.5
Chloroacetanilide herbicide	Alachlor[a]	420 (60)	806 (60)	38.8	1,421
Chloroacetanilide herbicide	Metolachlor[a]	326 (47)	626 (47)	44.8	1,550
Dinitroaniline herbicide	Pendimethalin[b]	264 (36)	521 (38)	8.8	501.7
Dinitroaniline herbicide	Trifluralin[a]	392 (56)	749 (56)	56	2,268
Imidazolinone herbicide	Imazethapyr[a]	278 (40)	542 (41)	20	661.5
Urea herbicide	Chlorimuron-ethyl[b]	243 (33)	415 (30)	8.8	392
Fungicide	Benomyl[b]	75 (10)	133 (10)	24.5	1,008
Fungicide	Captan[a]	71 (10)	157 (12)	0.3	27
Fungicide	Chlorothalonil[a]	46 (6)	101 (7)	28	1,429.2
Fungicide	Maneb/Mancozeb[b]	69 (9)	133 (10)	24.5	1,344
Fungicide	Metalaxyl[b]	147 (20)	264 (19)	12.3	576
Fungicide	Ziram[b]	4 (0.6)	9 (0.7)	12.3	945
Fumigant	Aluminum Phosphide[b]	29 (4)	64 (5)	8	135
Fumigant	Carbon tetrachloride / carbon disulfide[b]	58 (8)	124 (9)	8	123
Fumigant	Ethylene Dibromide[b]	28 (4)	65 (5)	12.3	474

Supplemental Material, Table 1 (cont.)

Chemical/functional class	Pesticide[a,b]	Prevalence of use (% Ever[c]) Cases n (%)	Control n (%)	Median lifetime days[d]	Median intensity-weighted lifetime days[d]
Fumigant	Methyl Bromide[a]	116 (15)	212 (15)	25.5	784
Other	Petroleum oil/petroleum distillate[b]	237 (33)	395 (29)	24.5	1,095

Abbreviations: OC, organochlorine insecticide; OP, organophosphate insecticide.
[a]Lifetime days and intensity-weighted lifetime days computed from information provided on enrollment questionnaire.
[b]Lifetime days and intensity-weighted lifetime days computed from information provided on take home questionnaire.
[c]Among participants not missing data for ever/never exposure to the given pesticide.
[d]Determined based on distributions of lifetime days and intensity-weighted lifetime days of application among exposed controls.

Supplemental Material, Table 2. BER gene SNPs that showed no effect on prostate cancer (p_{trend}>=0.05) in the Agricultural Health Study.

SNP	Gene	Variant (effect) allele	Wild-type allele	Chromosome	MAF[a]	OR[b]	L95[b]	U95[b]	p_{trend}[b]
rs7306515	*TDG*	T	A	12	0.37	1.14	1.00	1.29	0.05
rs304725	*XRCC1*	C	G	19	0.48	0.88	0.78	1.00	0.05
rs4135150	*TDG*	C	T	12	0.11	0.81	0.66	1.00	0.05
rs812498	*TDG*	C	T	12	0.21	0.86	0.74	1.01	0.06
rs6830266	*NEIL3*	A	G	4	0.17	0.85	0.71	1.01	0.06
rs7136550	*TDG*	C	G	12	0.37	1.13	0.99	1.29	0.06
rs1713460	*APEX1*	G	A	14	0.28	1.14	0.99	1.31	0.06
rs1713459	*APEX1*	T	C	14	0.22	1.14	0.99	1.33	0.07
rs3757440	*NUDT1*	G	A	7	0.35	1.12	0.99	1.28	0.07
rs4721505	*NUDT1*	A	G	7	0.31	0.89	0.77	1.01	0.08
rs7317188	*PARP4*	G	C	13	0.34	1.12	0.99	1.28	0.08
rs6539116	*TDG*	T	G	12	0.35	1.12	0.98	1.27	0.09
rs6600233	*MPG*	T	C	16	0.43	1.11	0.98	1.26	0.09
rs3219142	*PARP1*	A	G	1	0.21	0.88	0.75	1.03	0.10
rs3219243	*UNG*	C	T	12	0.19	1.14	0.98	1.33	0.10
rs7978946	*UNG,ALKBH2*	T	C	12	0.21	1.13	0.97	1.31	0.11
rs1165693	*TDG*	A	G	12	0.31	0.89	0.78	1.02	0.11
rs2700505	*TDG*	C	T	12	0.31	0.90	0.78	1.02	0.11
rs12446117	*NTHL1,TSC2*	G	A	16	0.22	1.13	0.97	1.30	0.11
rs2153609	*MUTYH*	G	A	1	0.24	1.12	0.97	1.29	0.12
rs11853141	*NEIL1*	T	C	15	0.44	1.10	0.98	1.25	0.12
rs10470431	*MBD4*	G	A	3	0.14	1.15	0.97	1.36	0.12
rs1054875	*POLG*	T	A	15	0.37	1.10	0.97	1.25	0.12
rs9994906	*NEIL3*	T	C	4	0.12	0.85	0.70	1.05	0.13
rs4135066	*TDG*	C	T	12	0.28	0.89	0.77	1.03	0.13
rs2569987	*UNG,ALKBH2*	C	T	12	0.18	0.88	0.75	1.04	0.13
rs2185549	*MUTYH*	C	T	1	0.24	1.12	0.97	1.29	0.13

Supplemental Material, Table 2 (cont.)

SNP	Gene	Variant (effect) allele	Wild-type allele	Chromosome	MAF[a]	OR[b]	L95[b]	U95[b]	p_{trend}[b]
rs8017603	*APEX1*	T	C	14	0.50	0.91	0.81	1.03	0.14
rs2298018	*MUTYH*	C	T	1	0.24	1.11	0.97	1.28	0.14
rs174548	*FEN1*	G	C	11	0.29	0.90	0.78	1.04	0.14
rs4688850	*MBD4*	T	C	3	0.13	0.87	0.72	1.05	0.15
rs750771	*PARP4*	T	C	13	0.19	1.12	0.96	1.30	0.15
rs1983132	*NEIL3*	T	C	4	0.13	0.87	0.72	1.05	0.15
rs1859248	*LIG3*	A	G	17	0.10	0.86	0.69	1.06	0.16
rs4273365	*MBD4*	C	T	3	0.13	1.13	0.95	1.35	0.16
rs1807271	*PARP4*	T	C	13	0.23	0.89	0.76	1.04	0.16
rs2271347	*PARP1*	A	G	1	0.21	1.11	0.96	1.28	0.16
rs8679	*PARP1*	G	A	1	0.21	1.11	0.96	1.28	0.16
rs3828486	*NEIL3*	G	T	4	0.15	1.13	0.95	1.33	0.16
rs2351000	*POLG*	G	A	15	0.13	0.88	0.73	1.06	0.16
rs2074520	*LIG3*	C	G	17	0.10	0.86	0.69	1.06	0.17
rs1888869	*APTX*	C	G	9	0.47	0.91	0.81	1.04	0.17
rs11609456	*POLE*	C	T	12	0.11	0.87	0.71	1.07	0.18
rs3219110	*PARP1*	C	T	1	0.49	0.92	0.82	1.04	0.18
rs163100	*UNG2*	A	C	5	0.18	1.11	0.95	1.30	0.18
rs12143101	*PARP1*	C	G	1	0.15	1.12	0.95	1.33	0.19
rs1351554	*LIG3*	T	C	17	0.12	0.88	0.72	1.07	0.19
rs2283430	*POLG*	A	C	15	0.37	1.09	0.96	1.24	0.19
rs10971263	*APTX*	G	A	9	0.11	1.14	0.94	1.37	0.19
rs938891	*APEX1*	C	G	14	0.34	0.92	0.8	1.05	0.20
rs12878052	*APEX1*	C	T	14	0.30	0.91	0.8	1.05	0.20
rs11624126	*APEX1*	C	T	14	0.35	1.09	0.96	1.25	0.20
rs8180912	*NEIL2*	T	C	8	0.19	1.11	0.95	1.29	0.20
rs34259	*UNG,ALKBH2*	C	G	12	0.20	1.11	0.95	1.29	0.20

Supplemental Material, Table 2 (cont.)

SNP	Gene	Variant (effect) allele	Wild-type allele	Chromosome	MAF[a]	OR[b]	L95[b]	U95[b]	p_{trend}[b]
rs2066505	*LIG3*	A	G	17	0.10	0.87	0.70	1.08	0.20
rs1673041	*POLD1*	T	G	19	0.24	0.91	0.78	1.05	0.20
rs758130	*POLG*	G	A	15	0.37	1.09	0.96	1.23	0.20
rs3176238	*POLG*	T	C	15	0.37	1.09	0.96	1.23	0.20
rs34263	*UNG*	G	A	12	0.20	1.10	0.95	1.28	0.21
rs159157	*OGG1*	C	T	3	0.11	0.88	0.71	1.08	0.21
rs3219104	*PARP1*	A	C	1	0.17	1.11	0.94	1.31	0.21
rs16943011	*POLG*	G	T	15	0.37	1.08	0.96	1.23	0.21
rs5744990	*POLE*	A	G	12	0.16	0.9	0.76	1.07	0.22
rs12436475	*APEX1*	A	T	14	0.11	0.88	0.72	1.08	0.22
rs163098	*UNG2*	T	C	5	0.10	1.13	0.93	1.37	0.23
rs2686187	*NEIL2*	A	G	8	0.42	1.08	0.95	1.22	0.23
rs108499	*FEN1*	T	C	11	0.34	0.92	0.81	1.05	0.23
rs2353005	*PNKP*	A	G	19	0.15	0.90	0.75	1.07	0.23
rs1983130	*NEIL3*	G	T	4	0.41	1.08	0.95	1.22	0.23
rs6490945	*PARP4*	C	T	13	0.29	1.08	0.95	1.24	0.24
rs11614717	*POLE*	T	G	12	0.17	0.90	0.76	1.07	0.24
rs938889	*APEX1*	T	C	14	0.40	0.93	0.81	1.05	0.24
rs2307438	*POLG*	C	A	15	0.37	1.08	0.95	1.22	0.24
rs2238300	*POLG*	A	G	15	0.37	1.08	0.95	1.22	0.24
rs17064576	*NEIL3*	T	C	4	0.16	0.90	0.76	1.07	0.25
rs2240576	*NUDT1*	A	C	7	0.24	0.92	0.79	1.06	0.25
rs2083919	*PCNA*	C	T	20	0.11	0.88	0.72	1.09	0.25
rs804256	*NEIL2*	C	T	8	0.35	1.08	0.95	1.22	0.25
rs10774961	*UNG,ALKBH2*	G	A	12	0.49	1.08	0.95	1.22	0.25
rs2877985	*NEIL3*	G	A	4	0.11	1.12	0.92	1.36	0.26
rs334888	*UNG2*	A	C	5	0.15	1.10	0.93	1.30	0.26

Supplemental Material, Table 2 (cont.)

SNP	Gene	Variant (effect) allele	Wild-type allele	Chromosome	MAF[a]	OR[b]	L95[b]	U95[b]	p_{trend}[b]
rs159154	*OGG1*	T	C	3	0.29	1.08	0.94	1.24	0.26
rs3093930	*PARP2*	T	C	14	0.30	0.92	0.81	1.06	0.26
rs11160682	*APEX1*	G	A	14	0.36	1.08	0.95	1.23	0.26
rs1062492	*NUDT1*	T	C	7	0.20	0.91	0.78	1.07	0.26
rs1138465	*POLG*	C	T	15	0.37	1.08	0.95	1.22	0.26
rs976072	*POLG*	G	A	15	0.37	1.08	0.95	1.22	0.26
rs938890	*APEX1*	A	G	14	0.40	0.93	0.82	1.06	0.26
rs1317996	*SMUG1*	C	G	12	0.49	1.07	0.95	1.21	0.27
rs2135617	*NEIL3*	G	T	4	0.37	0.93	0.82	1.06	0.27
rs10421339	*LIG1*	C	G	19	0.49	1.07	0.95	1.21	0.27
rs7984513	*PARP4*	A	G	13	0.46	0.93	0.82	1.06	0.28
rs2012359	*PARP4*	G	C	13	0.13	1.10	0.92	1.32	0.28
rs13250578	*NEIL2*	T	A	8	0.13	1.10	0.92	1.32	0.28
rs12432116	*APEX1*	T	G	14	0.11	0.89	0.73	1.10	0.28
rs10971333	*APTX*	C	T	9	0.11	1.11	0.91	1.35	0.29
rs125700	*OGG1*	C	A	3	0.12	0.9	0.74	1.10	0.30
rs9329248	*NEIL2*	A	C	8	0.18	0.92	0.78	1.08	0.30
rs704145	*APEX2*	T	C	23	0.19	1.12	0.90	1.40	0.30
rs6580978	*SMUG1*	A	G	12	0.45	1.07	0.94	1.21	0.30
rs7963858	*POLE*	T	C	12	0.32	1.07	0.94	1.23	0.30
rs159159	*OGG1*	C	A	3	0.30	1.07	0.94	1.23	0.30
rs2562148	*MPG*	C	A	16	0.34	1.07	0.94	1.22	0.31
rs11842915	*PARP4*	T	C	13	0.17	1.09	0.92	1.28	0.31
rs2271343	*PARP1*	G	C	1	0.17	0.92	0.77	1.09	0.31
rs10131831	*APEX1*	A	G	14	0.25	0.93	0.81	1.07	0.31
rs17675452	*NEIL3*	T	G	4	0.22	0.92	0.79	1.08	0.31
rs809202	*NEIL2*	C	T	8	0.41	1.07	0.94	1.21	0.32

Supplemental Material, Table 2 (cont.)

SNP	Gene	Variant (effect) allele	Wild-type allele	Chromosome	MAF[a]	OR[b]	L95[b]	U95[b]	p_{trend}[b]
rs9507365	*PARP4*	A	G	13	0.27	0.93	0.81	1.07	0.33
rs10813912	*APTX*	A	G	9	0.38	1.07	0.94	1.21	0.33
rs274876	*LIG1*	A	G	19	0.39	0.94	0.83	1.07	0.33
rs7150924	*APEX1*	G	A	14	0.43	1.06	0.94	1.21	0.33
rs1002153	*PARP1*	C	T	1	0.17	0.92	0.78	1.09	0.33
rs3219095	*PARP1*	C	T	1	0.16	1.09	0.92	1.28	0.33
rs3087404	*SMUG1*	T	C	12	0.45	1.06	0.94	1.20	0.33
rs10009807	*NEIL3*	A	G	4	0.28	1.07	0.93	1.23	0.33
rs2255403	*PARP1*	G	A	1	0.16	1.09	0.92	1.29	0.33
rs1062219	*NEIL2*	T	C	8	0.44	1.06	0.94	1.21	0.33
rs740006	*FEN1*	C	T	11	0.10	0.90	0.74	1.11	0.34
rs12501127	*NEIL3*	A	G	4	0.21	1.08	0.93	1.25	0.34
rs6107581	*PCNA*	C	A	20	0.11	0.90	0.74	1.11	0.34
rs17111750	*APEX1*	T	C	14	0.31	1.07	0.93	1.22	0.34
rs159153	*OGG1*	C	T	3	0.31	1.07	0.93	1.22	0.34
rs7140314	*APEX1*	G	C	14	0.39	0.94	0.82	1.07	0.34
rs231622	*UNG2*	A	G	5	0.27	1.07	0.93	1.23	0.35
rs4429194	*APEX1*	G	C	14	0.40	0.94	0.82	1.07	0.35
rs1045001	*MPG*	T	G	16	0.17	1.08	0.92	1.27	0.35
rs1136410	*PARP1*	G	A	1	0.16	1.08	0.92	1.28	0.35
rs6600227	*MPG*	C	A	16	0.34	0.94	0.81	1.08	0.35
rs6053149	*PCNA*	T	A	20	0.49	0.94	0.83	1.07	0.35
rs7531668	*PARP1*	T	A	1	0.17	0.92	0.78	1.09	0.36
rs804266	*NEIL2*	T	A	8	0.42	1.06	0.94	1.20	0.36
rs2562182	*MPG*	G	A	16	0.15	0.92	0.77	1.10	0.36
rs17277375	*APEX1*	T	C	14	0.34	1.06	0.93	1.22	0.36
rs752307	*PARP1*	C	G	1	0.16	1.08	0.91	1.28	0.36

Supplemental Material, Table 2 (cont.)

SNP	Gene	Variant (effect) allele	Wild-type allele	Chromosome	MAF[a]	OR[b]	L95[b]	U95[b]	p_{trend}[b]
rs1018783	*UNG,ALKBH2*	A	T	12	0.16	1.08	0.92	1.27	0.36
rs9511308	*PARP4*	T	A	13	0.24	0.93	0.81	1.08	0.37
rs3805170	*NEIL3*	A	G	4	0.41	1.06	0.93	1.20	0.37
rs8191534	*NEIL2*	T	A	8	0.25	0.94	0.81	1.08	0.37
rs274873	*LIG1*	T	C	19	0.50	0.95	0.84	1.07	0.38
rs10021940	*NEIL3*	A	G	4	0.22	0.93	0.80	1.09	0.38
rs13262643	*NEIL2*	C	G	8	0.15	0.92	0.77	1.10	0.38
rs4802703	*POLD1*	A	C	19	0.31	1.06	0.93	1.22	0.38
rs867612	*NEIL3*	A	G	4	0.26	1.07	0.92	1.23	0.38
rs11083918	*LIG1*	A	G	19	0.48	1.06	0.93	1.20	0.39
rs2288490	*MPG*	T	C	16	0.33	0.94	0.82	1.08	0.39
rs1052133	*OGG1*	G	C	3	0.23	0.94	0.81	1.09	0.39
rs9581094	*PARP4*	C	T	13	0.16	1.07	0.91	1.26	0.39
rs5745001	*POLE*	C	T	12	0.40	1.06	0.93	1.20	0.41
rs2293618	*TDG*	T	C	12	0.21	0.94	0.80	1.10	0.41
rs2645447	*NEIL2*	G	C	8	0.22	1.06	0.92	1.23	0.41
rs2010628	*NEIL2*	T	G	8	0.24	0.94	0.81	1.09	0.42
rs10161263	*SMUG1*	T	C	12	0.32	1.06	0.93	1.21	0.42
rs2074969	*NTHL1,TSC2*	G	A	16	0.49	1.05	0.93	1.19	0.42
rs4759345	*SMUG1*	C	T	12	0.41	1.05	0.93	1.19	0.42
rs4465523	*APEX1*	A	G	14	0.34	1.06	0.92	1.20	0.43
rs12568297	*PARP1*	C	G	1	0.37	0.95	0.83	1.08	0.43
rs2077197	*PARP1*	T	C	1	0.16	0.93	0.78	1.11	0.43
rs13264774	*NEIL2*	T	C	8	0.17	0.94	0.79	1.11	0.44
rs11784693	*NEIL2*	T	C	8	0.28	1.06	0.92	1.21	0.44
rs3809549	*NEIL1*	A	G	15	0.50	1.05	0.93	1.19	0.44
rs12050102	*APEX1*	T	G	14	0.34	1.05	0.92	1.20	0.44

Supplemental Material, Table 2 (cont.)

SNP	Gene	Variant (effect) allele	Wild-type allele	Chromosome	MAF[a]	OR[b]	L95[b]	U95[b]	p_{trend}[b]
rs4883627	*POLE*	T	C	12	0.40	1.05	0.93	1.19	0.44
rs10958713	*POLB*	T	C	8	0.37	0.95	0.84	1.08	0.44
rs16981519	*LIG1*	T	C	19	0.36	0.95	0.83	1.08	0.44
rs10418141	*PNKP*	A	C	19	0.30	1.05	0.92	1.21	0.44
rs2074885	*POLG*	A	G	15	0.15	1.07	0.90	1.26	0.44
rs274869	*LIG1*	G	A	19	0.48	1.05	0.93	1.19	0.44
rs2072668	*OGG1*	G	C	3	0.23	0.94	0.81	1.10	0.45
rs2516734	*NTHL1,TSC2*	G	C	16	0.13	0.93	0.77	1.12	0.45
rs10423897	*PNKP*	C	T	19	0.35	1.05	0.92	1.19	0.46
rs1889363	*PARP2*	A	G	14	0.34	0.95	0.83	1.09	0.46
rs4239761	*PCNA*	G	A	20	0.21	1.06	0.91	1.23	0.46
rs2160603	*UNG*	C	T	12	0.17	1.06	0.9	1.25	0.47
rs1001581	*XRCC1*	T	C	19	0.38	1.05	0.92	1.19	0.47
rs9507362	*PARP4*	C	A	13	0.29	0.95	0.82	1.09	0.47
rs9635769	*LIG3*	C	T	17	0.42	0.96	0.84	1.08	0.47
rs5744873	*POLE*	T	G	12	0.40	1.05	0.92	1.19	0.47
rs3757949	*NEIL2*	C	G	8	0.25	0.95	0.82	1.10	0.48
rs2074968	*NTHL1,TSC2*	C	G	16	0.42	0.96	0.84	1.08	0.48
rs7182283	*NEIL1*	G	T	15	0.50	0.96	0.85	1.08	0.48
rs412334	*FEN1*	T	C	11	0.15	1.06	0.9	1.26	0.48
rs7159947	*PARP2*	C	T	14	0.35	0.95	0.83	1.09	0.48
rs2645399	*NEIL2*	T	C	8	0.33	1.05	0.92	1.19	0.48
rs3730912	*LIG1*	T	G	19	0.12	0.94	0.78	1.13	0.49
rs11623831	*PARP2*	A	T	14	0.34	0.95	0.84	1.09	0.49
rs4841588	*NEIL2*	T	G	8	0.15	0.94	0.79	1.12	0.49
rs1637728	*NUDT1*	G	T	7	0.36	0.96	0.84	1.09	0.49
rs6814204	*NEIL3*	T	A	4	0.31	1.05	0.92	1.20	0.49

Supplemental Material, Table 2 (cont.)

SNP	Gene	Variant (effect) allele	Wild-type allele	Chromosome	MAF[a]	OR[b]	L95[b]	U95[b]	p_{trend}[b]
rs6490943	*PARP4*	G	C	13	0.18	1.06	0.9	1.24	0.49
rs12645561	*NEIL3*	T	C	4	0.13	1.07	0.89	1.28	0.49
rs8191604	*NEIL2*	G	T	8	0.27	0.95	0.83	1.10	0.50
rs12610706	*PNKP*	G	A	19	0.15	0.94	0.79	1.12	0.50
rs2048073	*NEIL3*	A	G	4	0.19	0.95	0.81	1.11	0.50
rs9578753	*PARP4*	T	C	13	0.12	1.07	0.89	1.28	0.50
rs10915989	*PARP1*	A	G	1	0.25	1.05	0.91	1.21	0.50
rs10007075	*NEIL3*	T	G	4	0.24	0.95	0.82	1.10	0.50
rs17791802	*NUDT1*	G	A	7	0.24	0.95	0.82	1.10	0.51
rs2275660	*PARP4*	C	T	13	0.23	1.05	0.91	1.21	0.51
rs746738	*PNKP*	C	T	19	0.42	0.96	0.85	1.09	0.51
rs2073636	*NTHL1,TSC2*	A	G	16	0.38	0.96	0.84	1.09	0.52
rs2074519	*LIG3*	T	C	17	0.42	1.04	0.92	1.19	0.52
rs1489953	*NEIL3*	G	C	4	0.47	1.04	0.92	1.19	0.52
rs11622655	*PARP2*	G	A	14	0.26	0.95	0.83	1.10	0.52
rs750391	*PARP4*	T	C	13	0.34	0.96	0.84	1.09	0.52
rs2645400	*NEIL2*	G	T	8	0.34	0.96	0.84	1.09	0.52
rs2244095	*POLD1*	A	G	19	0.11	0.94	0.76	1.15	0.52
rs3213282	*XRCC1*	G	C	19	0.47	0.96	0.85	1.09	0.52
rs2723877	*TDG*	T	C	12	0.11	1.07	0.87	1.30	0.54
rs17153747	*NEIL2*	C	T	8	0.12	0.94	0.78	1.14	0.54
rs1760944	*APEX1*	T	G	14	0.40	0.96	0.84	1.09	0.54
rs12863638	*PARP4*	A	C	13	0.24	0.95	0.82	1.11	0.54
rs11616577	*PARP4*	T	G	13	0.10	1.06	0.87	1.30	0.54
rs882383	*SMUG1*	A	G	12	0.11	1.06	0.87	1.29	0.55
rs140695	*MBD4*	T	C	3	0.29	0.96	0.84	1.10	0.55
rs10971316	*APTX*	A	G	9	0.42	1.04	0.92	1.18	0.56

Supplemental Material, Table 2 (cont.)

SNP	Gene	Variant (effect) allele	Wild-type allele	Chromosome	MAF[a]	OR[b]	L95[b]	U95[b]	p_{trend}[b]
rs1274517	*POLD1*	G	A	19	0.33	1.04	0.91	1.19	0.56
rs2248995	*TDG*	A	G	12	0.11	1.06	0.87	1.29	0.56
rs7682807	*NEIL3*	G	C	4	0.20	0.96	0.82	1.12	0.56
rs8019594	*APEX1*	G	T	14	0.35	0.96	0.84	1.10	0.57
rs10870494	*POLE*	T	C	12	0.44	1.04	0.92	1.17	0.57
rs938881	*APEX1*	A	T	14	0.41	0.96	0.85	1.10	0.57
rs2686184	*NEIL2*	A	G	8	0.41	1.04	0.92	1.17	0.57
rs3786763	*LIG1*	A	G	19	0.12	0.95	0.78	1.15	0.58
rs12485319	*MBD4*	G	A	3	0.14	0.95	0.79	1.14	0.58
rs1061438	*MPG*	A	G	16	0.14	0.95	0.79	1.14	0.58
rs10012298	*NEIL3*	A	T	4	0.14	0.95	0.79	1.14	0.58
rs11834788	*SMUG1*	G	A	12	0.46	1.04	0.92	1.17	0.58
rs1274513	*POLD1*	C	G	19	0.33	1.04	0.91	1.18	0.58
rs2247233	*POLG*	T	C	15	0.47	1.04	0.91	1.17	0.59
rs4796030	*LIG3*	A	C	17	0.43	1.04	0.91	1.18	0.59
rs1878705	*APEX1*	G	A	14	0.39	0.97	0.85	1.10	0.59
rs2302071	*NUDT1*	A	C	7	0.34	0.96	0.84	1.10	0.59
rs12026893	*MUTYH*	G	A	1	0.13	0.95	0.79	1.15	0.60
rs3745516	*POLD1*	A	G	19	0.23	1.04	0.90	1.20	0.60
rs3744358	*LIG3*	G	T	17	0.33	0.97	0.85	1.10	0.60
rs2046516	*SMUG1*	A	G	12	0.11	1.05	0.87	1.28	0.60
rs4769352	*PARP4*	A	G	13	0.38	0.97	0.85	1.10	0.61
rs867858	*NEIL2*	C	A	8	0.31	0.97	0.85	1.10	0.61
rs3211994	*NTHL1,TSC2*	T	C	16	0.19	1.04	0.89	1.22	0.62
rs2516740	*NTHL1,TSC2*	C	A	16	0.19	1.04	0.89	1.23	0.62
rs4883536	*POLE*	T	C	12	0.30	1.04	0.90	1.19	0.62
rs7966201	*SMUG1*	C	T	12	0.31	0.97	0.84	1.11	0.63

Supplemental Material, Table 2 (cont.)

SNP	Gene	Variant (effect) allele	Wild-type allele	Chromosome	MAF[a]	OR[b]	L95[b]	U95[b]	p_{trend}[b]
rs1130409	*APEX1*	G	T	14	0.49	1.03	0.91	1.17	0.63
rs2686206	*NEIL2*	T	C	8	0.45	1.03	0.91	1.17	0.64
rs2248949	*POLD1*	A	G	19	0.41	1.03	0.9	1.18	0.64
rs3818938	*PARP4*	A	G	13	0.40	0.97	0.85	1.10	0.65
rs758131	*POLG*	A	T	15	0.47	1.03	0.91	1.16	0.65
rs2029166	*SMUG1*	T	C	12	0.26	1.03	0.90	1.19	0.65
rs7160770	*PARP2*	C	T	14	0.48	0.97	0.86	1.10	0.65
rs251693	*LIG1*	C	T	19	0.45	1.03	0.91	1.17	0.66
rs12505306	*NEIL3*	T	A	4	0.12	1.04	0.86	1.26	0.66
rs6677172	*PARP1*	G	C	1	0.42	0.97	0.86	1.10	0.66
rs4883582	*POLE*	A	C	12	0.13	0.96	0.8	1.15	0.66
rs2048075	*NEIL3*	G	A	4	0.45	1.03	0.91	1.17	0.66
rs3729558	*PCNA*	C	G	20	0.47	0.97	0.86	1.10	0.67
rs3120073	*APEX1*	C	T	14	0.26	1.03	0.89	1.19	0.67
rs3810378	*XRCC1*	C	G	19	0.35	1.03	0.90	1.17	0.67
rs17754589	*NEIL2*	T	C	8	0.23	0.97	0.84	1.12	0.68
rs3730872	*LIG1*	A	G	19	0.11	0.96	0.79	1.17	0.68
rs1013358	*MPG*	C	T	16	0.13	1.04	0.87	1.24	0.69
rs3213334	*XRCC1*	A	G	19	0.25	0.97	0.84	1.12	0.69
rs2290775	*PNKP*	T	C	19	0.40	0.97	0.86	1.11	0.69
rs9511316	*PARP4*	A	G	13	0.31	1.03	0.90	1.17	0.69
rs17064578	*NEIL3*	C	T	4	0.11	0.96	0.79	1.17	0.69
rs2063060	*NEIL3*	C	G	4	0.25	1.03	0.89	1.19	0.70
rs6426551	*PARP1*	A	G	1	0.25	1.03	0.89	1.19	0.70
rs3176208	*POLG*	G	T	15	0.10	0.96	0.78	1.18	0.70
rs159146	*OGG1*	A	G	3	0.26	1.03	0.89	1.19	0.70
rs2516781	*NTHL1,TSC2*	T	C	16	0.31	0.97	0.84	1.12	0.70

Supplemental Material, Table 2 (cont.)

SNP	Gene	Variant (effect) allele	Wild-type allele	Chromosome	MAF[a]	OR[b]	L95[b]	U95[b]	p_{trend}[b]
rs804280	*NEIL2*	C	A	8	0.40	1.03	0.90	1.16	0.70
rs11611943	*SMUG1*	G	A	12	0.19	0.97	0.83	1.13	0.70
rs938883	*APEX1*	T	C	14	0.45	0.98	0.86	1.11	0.70
rs10971259	*APTX*	T	C	9	0.14	1.04	0.86	1.24	0.71
rs13273672	*NEIL2*	C	T	8	0.33	0.98	0.86	1.11	0.71
rs176641	*POLG*	C	A	15	0.35	0.98	0.86	1.11	0.71
rs3135998	*LIG3*	A	G	17	0.40	1.02	0.90	1.17	0.71
rs5744944	*POLE*	C	T	12	0.45	1.02	0.90	1.16	0.72
rs334879	*UNG2*	A	G	5	0.14	1.03	0.87	1.23	0.72
rs17090614	*NEIL3*	A	G	4	0.10	0.96	0.79	1.18	0.73
rs2288878	*LIG1*	T	C	19	0.45	1.02	0.90	1.16	0.73
rs3730924	*LIG1*	T	C	19	0.38	0.98	0.86	1.11	0.74
rs3093942	*PARP2,TEP1*	C	A	14	0.20	0.97	0.83	1.14	0.74
rs8003245	*APEX1*	T	A	14	0.38	1.02	0.90	1.16	0.74
rs17726200	*NEIL3*	G	T	4	0.30	0.98	0.85	1.12	0.74
rs1713419	*PARP2,TEP1*	G	A	14	0.44	0.98	0.86	1.11	0.75
rs2163619	*LIG1*	G	A	19	0.49	0.98	0.87	1.11	0.75
rs3751209	*TDG*	A	G	12	0.22	1.02	0.88	1.19	0.76
rs2386523	*LIG1*	C	T	19	0.50	0.98	0.87	1.11	0.76
rs6560896	*POLE*	T	C	12	0.44	1.02	0.90	1.15	0.76
rs3211995	*NTHL1,TSC2*	A	G	16	0.17	1.03	0.87	1.21	0.76
rs9920768	*POLG*	C	G	15	0.46	1.02	0.90	1.15	0.76
rs8015748	*APEX1*	T	C	14	0.38	1.02	0.90	1.16	0.76
rs8063461	*NTHL1,TSC2*	A	G	16	0.39	0.98	0.86	1.11	0.77
rs4981998	*PARP2,TEP1*	T	C	14	0.21	1.02	0.88	1.20	0.77
rs2074518	*LIG3*	T	C	17	0.46	1.02	0.90	1.16	0.77
rs999692	*APEX1*	C	T	14	0.34	1.02	0.89	1.16	0.77

Supplemental Material, Table 2 (cont.)

SNP	Gene	Variant (effect) allele	Wild-type allele	Chromosome	MAF[a]	OR[b]	L95[b]	U95[b]	p_{trend}[b]
rs7153128	*APEX1*	T	A	14	0.26	1.02	0.89	1.18	0.78
rs4883577	*POLE*	A	T	12	0.45	1.02	0.90	1.15	0.78
rs3135967	*LIG3*	G	A	17	0.48	1.02	0.90	1.15	0.78
rs2682587	*XRCC1*	A	C	19	0.19	1.02	0.88	1.19	0.79
rs251690	*LIG1*	A	G	19	0.38	0.98	0.87	1.12	0.79
rs10915985	*PARP1*	T	C	1	0.42	0.98	0.87	1.11	0.79
rs2297617	*PARP2*	C	T	14	0.32	0.98	0.86	1.12	0.79
rs2319196	*APEX1*	A	G	14	0.26	1.02	0.88	1.18	0.79
rs11111854	*TDG*	G	A	12	0.14	1.02	0.86	1.22	0.81
rs2862909	*PARP4*	G	T	13	0.39	1.02	0.90	1.15	0.81
rs1290646	*PNKP*	A	G	19	0.48	0.99	0.87	1.11	0.81
rs4964435	*TDG*	T	G	12	0.14	1.02	0.86	1.22	0.81
rs875640	*POLG*	A	C	15	0.16	1.02	0.86	1.21	0.82
rs2275662	*PARP4*	T	C	13	0.11	0.98	0.80	1.19	0.82
rs939460	*XRCC1*	A	G	19	0.19	1.02	0.87	1.19	0.83
rs274883	*LIG1*	G	A	19	0.17	0.98	0.84	1.16	0.84
rs3784621	*DUT*	C	T	15	0.18	0.98	0.84	1.16	0.84
rs8017682	*APEX1*	A	G	14	0.14	0.98	0.82	1.18	0.84
rs731826	*PNKP*	G	T	19	0.42	0.99	0.87	1.12	0.85
rs17675654	*NEIL3*	G	C	4	0.10	1.02	0.83	1.25	0.85
rs1043180	*NEIL2*	T	C	8	0.12	0.98	0.81	1.19	0.85
rs3735816	*NEIL2*	T	C	8	0.50	1.01	0.89	1.15	0.85
rs11637235	*DUT*	C	T	15	0.23	0.99	0.85	1.14	0.85
rs12441867	*DUT*	T	C	15	0.16	1.02	0.86	1.20	0.86
rs7161611	*PARP2,TEP1*	A	C	14	0.26	0.99	0.86	1.14	0.86
rs2305922	*PNKP*	G	T	19	0.41	1.01	0.89	1.15	0.87
rs13068631	*PARP3*	C	G	3	0.34	1.01	0.89	1.15	0.87

Supplemental Material, Table 2 (cont.)

SNP	Gene	Variant (effect) allele	Wild-type allele	Chromosome	MAF[a]	OR[b]	L95[b]	U95[b]	p_{trend}[b]
rs3730913	*LIG1*	A	G	19	0.17	0.99	0.84	1.16	0.87
rs10147163	*PARP2,TEP1*	C	T	14	0.26	0.99	0.86	1.14	0.88
rs334882	*UNG2*	T	C	5	0.12	0.99	0.81	1.19	0.88
rs6982453	*NEIL2*	C	T	8	0.49	1.01	0.89	1.14	0.88
rs884368	*PARP2*	G	A	14	0.25	0.99	0.86	1.14	0.88
rs2854508	*XRCC1*	A	T	19	0.23	0.99	0.85	1.15	0.89
rs804292	*NEIL2*	G	A	8	0.24	1.01	0.87	1.17	0.89
rs16960758	*DUT*	C	T	15	0.16	0.99	0.83	1.18	0.89
rs3093882	*PARP2*	A	C	14	0.24	1.01	0.88	1.17	0.90
rs4981158	*PARP2,TEP1*	C	T	14	0.27	0.99	0.86	1.14	0.90
rs4770687	*PARP4*	G	A	13	0.41	1.01	0.89	1.14	0.90
rs2275008	*APEX1*	C	T	14	0.30	1.01	0.88	1.16	0.90
rs25487	*XRCC1*	T	C	19	0.36	1.01	0.88	1.15	0.91
rs804282	*NEIL2*	G	T	8	0.45	1.01	0.89	1.14	0.92
rs17108960	*SMUG1*	T	C	12	0.25	0.99	0.86	1.14	0.92
rs10407902	*LIG1*	G	C	19	0.12	0.99	0.82	1.20	0.92
rs804279	*NEIL2*	A	T	8	0.25	1.01	0.87	1.16	0.92
rs156640	*LIG1*	C	G	19	0.43	1.01	0.89	1.14	0.93
rs3093904	*PARP2*	A	T	14	0.24	1.01	0.87	1.16	0.94
rs4883537	*POLE*	A	G	12	0.44	1.01	0.89	1.14	0.94
rs11627273	*PARP2*	C	T	14	0.19	1.01	0.86	1.18	0.94
rs5744897	*POLE*	T	C	12	0.11	0.99	0.82	1.20	0.94
rs2249844	*PARP1*	C	T	1	0.33	1.00	0.87	1.14	0.94
rs11631385	*DUT*	G	A	15	0.17	0.99	0.84	1.17	0.94
rs3213356	*XRCC1*	C	T	19	0.45	1.00	0.89	1.14	0.95
rs2269112	*OGG1*	T	C	3	0.17	0.99	0.85	1.17	0.95
rs3093933	*PARP2*	T	G	14	0.24	1.00	0.87	1.16	0.95

Supplemental Material, Table 2 (cont.)

SNP	Gene	Variant (effect) allele	Wild-type allele	Chromosome	MAF[a]	OR[b]	L95[b]	U95[b]	p_{trend}[b]
rs10861148	*TDG*	A	C	12	0.10	0.99	0.81	1.22	0.95
rs8037626	*DUT*	G	A	15	0.16	1.00	0.84	1.19	0.96
rs175628	*LIG1*	G	A	19	0.38	1.00	0.88	1.13	0.97
rs7667508	*NEIL3*	T	C	4	0.46	1.00	0.89	1.14	0.97
rs6846091	*NEIL3*	G	C	4	0.29	1.00	0.87	1.15	0.97
rs293795	*OGG1*	G	A	3	0.19	1.00	0.86	1.18	0.97
rs13379705	*DUT*	C	T	15	0.16	1.00	0.85	1.19	0.97
rs3219337	*POLD1*	G	A	19	0.24	1.00	0.86	1.15	0.97
rs767540	*XRCC1*	T	C	19	0.14	1.00	0.83	1.19	0.98
rs804284	*NEIL2*	C	G	8	0.21	1.00	0.86	1.16	0.98
rs3784619	*DUT*	G	A	15	0.16	1.00	0.84	1.19	0.99
rs1866074	*TDG*	A	G	12	0.48	1.00	0.88	1.13	0.99
rs4840583	*NEIL2*	T	C	8	0.44	1.00	0.88	1.13	0.99
rs2700	*PARP2,TEP1*	C	A	14	0.28	1.00	0.87	1.15	0.99
rs10813916	*APTX*	C	T	9	0.24	1.00	0.87	1.15	0.99
rs509360	*FEN1*	A	G	11	0.32	1.00	0.87	1.14	0.99
rs7535487	*MUTYH*	A	T	1	0.10	1.00	0.82	1.23	1.00
rs12825	*NEIL2*	G	C	8	0.40	1.00	0.88	1.13	1.00
rs6846587	*NEIL3*	T	C	4	0.15	1.00	0.84	1.19	1.00
rs11131791	*NEIL3*	A	G	4	0.13	1.00	0.83	1.20	1.00
rs1104893	*PARP1*	G	A	1	0.33	1.00	0.88	1.14	1.00

Abbreviations: BER; base excision repair; L95 and U95, lower and upper bounds of 95% confidence interval, respectively; MAF, minor allele frequency; OR, odds ratio per allele; SNP, single nucleotide polymorphism.

[a]Among controls.

[b]Effect of variant allele using an ordinal SNP variable, assuming a log-additive genetic model and adjusting for age and state.

Supplemental Material, Table 3. BER genes included in the iSelect platform and the number of SNPs evaluated, interactions meeting FDR<0.2, and the most significant interaction by gene in the Agricultural Health Study.

Function	Gene	Number of SNPs evaluated	Effective number of SNPs[a]	Number of interactions with FDR < 0.2	Interactions with FDR<0.2	Pesticide x SNP combination with most significant interaction	p_{interact} for most significant interaction[b]	FDR *p*-value[c]
Glycosylase	*MBD4*	5	5	0	N/A	fonofos x rs4688850	4.5×10^{-3}	0.61
Glycosylase	*MPG*	8	7	0	N/A	glyphosate x rs1045001	3.7×10^{-3}	0.93
Glycosylase	*MUTYH* (*MYH*)	6	4	0	N/A	chlordane x rs7535487	2.7×10^{-3}	0.52
Glycosylase	*NEIL1*	3	2	0	N/A	EPTC x rs3809549	0.02	0.88
Glycosylase	*NEIL2*	35	27	0	N/A	dicamba x rs2686206	4.6×10^{-4}	0.63
Glycosylase	*NEIL3*	33	27	1	fonofos x rs1983132	fonofos x rs1983132	9.3×10^{-6}	0.01
Glycosylase	*NTHL1* (*NTH1*)	11	11	2	DDVP x rs8063461<br>terbufos x rs17654678	DDVP x rs8063461	7.0×10^{-4}	0.16
Glycosylase	*OGG1*	10	8	0	N/A	parathion x rs293795	4.7×10^{-3}	0.71
Glycosylase	*SMUG1*	12	10	0	N/A	glyphosate x rs4759345	3.4×10^{-3}	0.32
Glycosylase	*TDG*	20	10	0	N/A	parathion x rs4135150	9.2×10^{-4}	0.24
Glycosylase	*UNG*	11	8	0	N/A	butylate x rs10774961	0.02	0.99
Glycosylase	*UNG2*	7	5	0	N/A	metribuzin x rs163100	5.2×10^{-3}	0.68
Ligase	*LIG1*	20	9	0	N/A	terbufos x rs3786763	8.7×10^{-4}	0.51
Ligase	*LIG3*	11	7	0	N/A	phorate x rs9635769	0.01	0.94
Ligase accessory factor	*XRCC1*	13	10	0	N/A	fonofos x rs939460	6.0×10^{-4}	0.30
Polymerase-related	*PCNA*	5	5	0	N/A	glyphosate x rs2083919	1.4×10^{-3}	0.27
Polymerase	*POLB*	1	1	1	glyphosate x rs10958713	glyphosate x rs10958713	2.2×10^{-4}	8.2×10^{-3}
Polymerase	*POLD1*	8	7	0	N/A	carbaryl x rs3219337	4.3×10^{-3}	0.83
Polymerase	*POLE*	15	7	0	N/A	atrazine x rs5744897	9.6×10^{-4}	0.40
Polymerase	*POLG*	17	10	0	N/A	phorate x rs9920768	9.9×10^{-4}	0.20
Endonuclease	*APEX1* (*APE1*)	34	20	0	N/A	DDT x rs2319196	3.0×10^{-4}	0.28
Endonuclease	*APEX2* (*APE2*)	1	1	0	N/A	petroleum oil x rs704145	0.01	0.27

Supplemental Material, Table 3 (cont.)

Function	Gene	Number of SNPs evaluated	Effective number of SNPs[a]	Number of interactions with FDR < 0.2	Interactions with FDR<0.2	Pesticide x SNP combination with most significant interaction	$p_{interact}$ for most significant interaction[b]	FDR *p*-value[c]
Endonuclease	*FEN1* (*DNase IV*)	9	7	0	N/A	2,4,5-T x rs174532	$1.0x10^{-3}$	0.25
Single-stranded break repair	*APTX* (*aprataxin*)	7	5	0	N/A	metribuzin x rs10971333	$3.7x10^{-3}$	0.65
Modulation of nucleotide pools	*DUT*	8	6	10	carbaryl x rs11637235, rs11631385, rs3784619, rs13379705, rs16960758, rs8037626, rs12441867, rs3784621 malathion x rs11637235 diazinon x rs11637235	carbaryl x rs11637235	$1.3x10^{-5}$	$3.1x10^{-3}$
Modulation of nucleotide pools	*NUDT1* (*MTH1*)	10	8	0	N/A	methyl bromide x rs2240576	$2.6x10^{-3}$	0.97
Protection of strand interruptions	*PARP1* (*ADPRT*)	21	8	0	N/A	metribuzin x rs3219104	$8.4x10^{-4}$	0.57
Protection of strand interruptions	*PARP2* (*ADPRTL2*)	19	12	0	N/A	metalaxyl x rs1713419	$5.0x10^{-4}$	0.38
Protection of strand interruptions	*PARP3*	1	1	0	N/A	permethrin x rs13068631	0.01	0.49
Protection of strand interruptions	*PARP4*	23	16	0	N/A	carbaryl x rs7984513	$1.2x10^{-3}$	0.97
Conversion of breaks to ligatable ends	*PNKP*	10	9	0	N/A	phorate x rs10423897	$1.4x10^{-3}$	0.55

Abbreviations: BER, base excision repair; DDT, dichlorodiphenyltrichloroethane; DDVP, dichlorvos; EPTC, *S*-ethyl dipropylthiocarbamate; FDR, False Discovery Rate; SNP, single nucleotide polymorphism; 2,4,5-T, 2,4,5-trichlorophenoxyacetic acid.
[a]Effective number of SNPs after accounting for correlations between SNPs using the method described by Gao et al. (2008).
[b]*P*-value for interaction from LRT, treating pesticide exposure variables as ordinal variables, assuming the dominant genetic model, and adjusting for age and state.
[c]FDR-adjusted *p*-value for the most significant interaction.

Supplemental Material, Table 4. Interaction between *NEIL3* haplotypes and fonofos in the Agricultural Health Study.

NEIL3 haplotype[a]	Haplotype frequency among cases	Haplotype frequency among controls	Fonofos exposure	Interaction OR[b]	95% CI[b]	$p_{interact}$[b]
AGGACCT	0.62	0.62	None	REF	REF	
AGGACCC	0.03	0.03	Low	0.71	0.22- 2.27	0.56
			High	0.90	0.14- 6.00	0.92
AGGATCC	0.02	0.03	Low	1.03	0.24- 4.35	0.97
			High	5.90	1.21-28.77	**0.03**
AGGTTCT	0.02	0.02	Low	2.09	0.52-8.36	0.30
			High	1.91	0.38- 9.49	0.43
AGGTTCC	0.05	0.05	Low	1.67	0.63-4.47	0.31
			High	5.13	1.85-14.24	**1.7×10^{-3}**
AAATCTT	0.12	0.13	Low	0.85	0.43-1.67	0.63
			High	0.66	0.35- 1.27	0.22
TGGTTTT	0.02	0.03	Low	2.57	0.67-9.90	0.17
			High	3.05	0.94- 9.87	**0.06**
TGATCCT	0.10	0.08	Low	0.67	0.31- 1.45	0.31
			High	0.74	0.35- 1.58	0.44
Rare[c]	<0.01	<0.01	Low	0.57	0.10- 3.16	0.52
			High	3.05	0.54-17.14	0.21

Abbreviations: CI, 95% confidence interval; OR, odds ratio; SNP, single nucleotide polymorphism.
[a]SNP order: rs12505306, rs11131791, rs12501127, rs6814204, rs1983132, rs17064576, rs17064578.
[b]From the cross-product of the haplotype and the pesticide, adjusted for age and state, treating the pesticide as a categorical variable and assuming the additive model for haplotypes.
[c]Haplotypes with frequency<1%.

Supplemental Material, Table 5. Interaction between *TDG* haplotypes and terbufos in the Agricultural Health Study.

TDG haplotype[a]	Haplotype frequency among cases	Haplotype frequency among controls	Terbufos exposure	Interaction OR[b]	95% CI[b]	$p_{interact}$[b]
CGGCAGGATGCTTGTTGCGGGAGTGGTGTG	0.28	0.25	None	REF	REF	
CGGGAGAATGTATGTTACGGAAATGCTTGG	0.02	0.02	Low	0.27	0.05-1.43	0.12
			High	0.39	0.09-1.67	0.20
CGGGAGAATGTATGTTACGAGAATGGCGGG	0.10	0.11	Low	1.51	0.79-2.88	0.22
			High	1.39	0.74-2.60	0.31
CGGGAGAATGTATGTTACAGAAATGCTTGG	0.07	0.07	Low	1.06	0.51-2.23	0.87
			High	1.33	0.65-2.76	0.44
CGGGAAGATGCATGCTATGAGAATGGTGGG	0.11	0.11	Low	1.45	0.77-2.73	0.25
			High	1.07	0.56-2.03	0.85
CGGCAGGATGCTTGTTGCGGGAGTGGTGGG	0.03	0.02	Low	2.69	0.84-8.68	0.10
			High	1.57	0.46-5.35	0.47
CGGCAGGATGCTTGTTGCGGGAGCGGTGTG	0.06	0.06	Low	1.55	0.75-3.17	0.24
			High	2.27	1.03-5.02	**0.04**
CGGCAGGATGCTTGTTGCGGGCATGGTGTG	0.02	0.02	Low	1.80	0.49-6.53	0.37
			High	0.30	0.06-1.53	0.15
CAGGAGAACGCACACTACGAGAGTGGTGGG	0.14	0.16	Low	2.05	1.18-3.54	**0.01**
			High	2.16	1.21-3.88	**9.6×10^{-3}**
CAGGAGAGCGCACGTGACGGAAATGGTTGG	0.04	0.04	Low	1.28	0.48-3.37	0.62
			High	1.94	0.83-4.56	0.13
AAGGAGAGCGCATGTTACGGAAATGGTGGG	0.08	0.08	Low	1.28	0.65-2.53	0.47
			High	0.69	0.33-1.44	0.32
AAAGGGAGCTCATGTTACGAGCATAGTGGA	0.02	0.02	Low	1.42	0.28-7.13	0.67
			High	0.71	0.12-4.09	0.71
Rare[c]	<0.01	<0.01	Low	1.26	0.30-5.30	0.75
			High	1.45	0.37-5.77	0.60

Abbreviations: CI, 95% confidence interval; OR, odds ratio; SNP, single nucleotide polymorphism.

[a]SNP order: rs10861148, rs1165693, rs10507172, rs7136550, rs10507173, rs2248995, rs1047490, rs11111854, rs2700505, rs2293620, rs2293618, rs7306515, rs812498, rs322107, rs4135066, rs4135067, rs4135081, rs2723877, rs3751206, rs10861152, rs3751209, rs3829301, rs1866074, rs4135106, rs4135113, rs4135128, rs4135150, rs4964435, rs6539116, rs11111865.

[b]From the cross-product of the haplotype and the pesticide, adjusted for age and state, treating the pesticide as a categorical variable and assuming the additive model for haplotypes.

[c]Haplotypes with frequency<1%.

References

Gao X, Starmer J, Martin ER. 2008. A multiple testing correction method for genetic association studies using correlated single nucleotide polymorphisms. Genet Epidemiol 32(4):361-369.
